# Triphenyl‐Modified Mixed‐Mode Stationary Phases With and Without Embedded Ion‐Exchange Sites for High‐Performance Liquid Chromatography

**DOI:** 10.1002/jssc.70058

**Published:** 2024-12-23

**Authors:** Marc Wolter, Mirna Maalouf, Mateusz Janek, Cornelius Knappe, Markus Kramer, Michael Lämmerhofer

**Affiliations:** ^1^ Institute of Pharmaceutical Sciences, Pharmaceutical (Bio‐)Analysis University of Tübingen Tübingen Germany; ^2^ Institute of Organic Chemistry University of Tübingen Tübingen Germany

**Keywords:** mixed‐mode chromatography, oligonucleotide, silatrane, stationary phase, thiol‐yne/ene click reaction

## Abstract

The present work reports on the preparation, characterization, and evaluation of a set of novel triphenyl‐modified silica‐based stationary phases without and with embedded ion‐exchange sites for mixed‐mode liquid chromatography. The three synthesized triphenyl phases differed in additionally incorporated ion‐exchange sites. In one embodiment, allyltriphenylsilane was bonded to thiol‐modified silica by thiol‐ene click reaction, leading to particles with no ion‐exchange sites. A second stationary phase was obtained by thiol‐yne click reaction of thiol silica with 2‐propinyl‐triphenylphosphonium bromide, yielding a strong anion‐exchanger (SAX). A third stationary phase was obtained from this SAX phase by the oxidation of residual thiols to sulfonic acid moieties, leading to a zwitterionic surface. All synthesized materials were subjected to elemental analysis, ^13^C and ^29^Si solid‐state cross‐polarization/magic angle spinning nuclear magnetic resonance (CP/MAS NMR) spectroscopy analysis, and pH‐dependent ζ‐potential determinations via electrophoretic light scattering. The prepared stationary phases were chromatographically evaluated under classical reversed‐phase, ion‐exchange, and hydrophilic interaction chromatography conditions and classified within a set of commercially available columns by principal component analysis of retention factors. Finally, the obtained stationary phases were applied for biomolecule separations (e.g., teicoplanin and siRNA patisiran). These LC tests proved the orthogonality of the three prepared stationary phases and indicated possible fields of application.

**Abbreviations**
CP/MAS: cross‐polarization/magic angle spinning
MMC: mixed‐mode chromatography
siRNA: small interfering ribonucleic acid
SP: stationary phase
ZWIX: zwitterionic ion exchanger

## 1 Introduction

Nowadays, in biochemical and pharmaceutical analysis, a variety of diverse liquid chromatography modes are applied. Besides the most common reversed‐phase (RP) and hydrophilic interaction chromatography (HILIC) modes, mixed‐mode chromatography (MMC) is constantly getting more popular. Such MMC stationary phases offer two or more different types of interactions with the analyte and hence orthogonal retention mechanisms. Due to the complementarity of the interactions, distinct selectivities, more flexibility for optimization of separation conditions, and extended fields of applicability can usually be achieved than would be feasible with single‐mode chromatography. Therefore, hydrophobic, hydrophilic, and ionic interaction sites are most frequently combined with each other within one analytical column [1, 2, 3]. To this end, mixed beads, mixed ligands, or single multifunctional ligands strategies have been used to create the MMC character of the stationary phase [4]. The hydrophobic properties are typically incorporated by the implementation of alkyl moieties on the surface of the support, the hydrophilic character by embedding polar functional groups such as amide, urea, carbamate, ether or thioether moieties (as hydrogen acceptor or donor sites), whereas ionic interaction sites are usually introduced by the incorporation of permanently or pH‐dependent charged functional groups [2, 4–6]. Interestingly, aromatic groups, as for instance phenyl‐moieties, are rarely reported to be applied as the hydrophobic part of mixed‐mode phases [7, 8]. Some selected examples are typical ionic liquid phases that have immobilized, for example, imidazole‐type ionic liquids [9, 10, 11]. On the other hand, classical phenyl phases, including phenyl‐, biphenyl‐, phenylhexyl‐, and polyphenyl‐modified silica, reached attractiveness as alternative RP‐type stationary phases with complementary retention profiles when used with methanolic mobile phases that do not disrupt π‐π‐interactions like acetonitrile [12, 13, 14, 15, 16].

Phenyl groups alone have numerous other interaction options in addition to the hydrophobic ones, which makes them promising candidates for MMC phases. Thus, π─π‐, cation─π‐, anion─π‐, dipole─dipole‐, hydrogen‐bonding and weak electrostatic interactions are possible. In addition, the anchoring of such bulky phenyl groups to the silica surface can effectively shield free silanols and prevent detrimental analyte–silanol interactions. In particular, in the analysis of biopolymers, free and accessible silanol groups have been reported to be responsible for poor recoveries, badly shaped peaks, and low efficiencies. However, this phenomenon is usually largely solved by end capping strategies or the use of polymer‐based non‐silica supports [17, 18, 19, 20].

Against this backdrop, stationary phases with implemented bulky triphenyl residues imparting, on the one hand, RP chromatography properties and with embedded ionic moieties conferring, on the other hand, ion‐exchange properties were synthesized to create new RP/IEX MMC stationary phases. The latter was obtained by incorporation of permanently positively charged phosphonium groups and negatively charged sulfonic acid moieties on the surface. In contrast to sulfonic acid groups, only a few phosphonium‐type silica phases have been reported in liquid chromatography yet. A few permanently positively charged phosphonium‐functionalized stationary phases for HILIC of, for instance, phospholipids were reported, as well as stationary phases suitable for the analysis of inter alia lignin degradation products [21, 22, 23]. Phosphonium‐based ionic liquid stationary phases were mainly investigated in gas chromatography [24, 25, 26]. Furthermore, zwitterionic phases, including combined phosphonium, and sulfonic acid functionalities, were fabricated by Qui et al. and successfully applied for HILIC of inter alia nucleic acids, nucleosides, and water‐soluble vitamins [27]. In this study, the synthesis, characterization, and applicability of neutral, SAX‐type, and zwitterionic‐type triphenyl‐stationary phases with distinct surface charge will be briefly addressed.

## 2 Experimental

### 2.1 Materials

Spherical silica particles (Daisogel, 3 µm, 300 Å, 100 m^2^/g) were purchased from Daiso Fine Chem GmbH (Düsseldorf, Germany), and empty stainless‐steel columns (50 mm × 3 mm) were purchased from Bischoff Chromatography (Leonberg, Germany). Allyltriphenylsilane, *n*‐octadecyltrimethoxysilane, and *n*‐butyltrimethoxysilane were supplied from ABCR Chemicals (Karlsruhe, Germany). (3‐Mercaptopropyl)trimethoxysilane, 2‐propinyl‐triphenylphosphonium bromide, azobis (isobutyronitrile) (AIBN), 2,2′‐dipyridyl disulfide (DPDS), acetic acid (analytical grade), formic acid (FA, analytical grade), ammonium acetate, 4‐dimethylaminopyridine (DMAP), sodium hydroxide, deuterated chloroform, triethanolamine, butylbenzene (BuB), pentylbenzene (PeB), triphenylene, *o*‐terphenyl, pyridine, phenol, benzylamine, sodium *p*‐toluenesulfonate, adenosine, guanosine, cytidine, thymidine, uridine, nicotinic acid, pyridoxine hydrochloride, ascorbic acid, riboflavin, thiamine hydrochloride, caffeine, theophylline, theobromine, *O*,*O*‐diethylchlorothiophosphate (DECTP), triethylamine were received from Sigma‐Aldrich (Munich, Germany). *O*,*O*‐Diethylthiophosphate (DETP) was obtained from the hydrolysis of DECTP in the presence of triethylamine. (3‐Mercaptopropyl)silatrane (MPS) was synthesized as described in the Supporting Information (see Figures S1 and S2). The solvents toluene, methanol, and methylene chloride were of technical grade or HPLC grade and purchased from Brenntag (Essen, Germany) or Sigma‐Aldrich (Munich, Germany). MilliQ water was prepared by using an Elga PureLab Ultra Purification System (Celle, Germany).

### 2.2 Instrumentation and Software

Elemental analysis was conducted using an EA 3000 CHNS‐O elemental analyzer from EuroVector SpA (Milan, Italy) as described in [28, 29]. Determination of free and reactive sulfhydryl groups by a thiol‐disulfide exchange reaction using DPDS assay was carried out according to [29]. ζ‐Potentials were determined in accordance with [30] by electrophoretic light scattering measurements using a Zetasizer NanoZS particle analyzer from Malvern Instruments (Herrenberg, Germany). MarvinSketch 20.19 software (ChemAxon, www.chemaxon.de) was used to calculate pK_a_ values. Liquid‐state nuclear magnetic resonance (NMR) spectroscopy experiments were carried out using a Bruker Avance 400 MHz spectrometer (Bruker, Rheinstetten, Germany), whereas solid‐state NMR spectroscopy experiments were performed using a Bruker Avance III HD XWB 300 spectrometer (applied parameters can be found in [28]). NMR data were processed by Topspin 4.0.8 software from Bruker. HPLC experiments were all performed on HPLC systems from Agilent Technologies (Waldbronn, Germany). Thereby, measurements were usually performed using an Agilent 1260 series HPLC system featuring a degasser, flexible pump, autosampler, temperature‐controlled column compartment (TCC), and a diode array detector (DAD), or an Agilent 1100 series HPLC equipped with a degasser, quaternary pump, autosampler, TCC, and variable wavelength detector (VWD). OpenLab CDS ChemStation Online Software (Rev. C.01.03 (37), 2011) and ChemStation Offline Software (Rev. B.04.03 (16), 2010) from Agilent Technologies were used for data acquisition and analysis. Stationary phases were slurry packed into stainless steel columns using a Smartline Pneumatic Pump 1950 from Knauer (Berlin, Germany) and a packing apparatus obtained from Dr. Maisch HPLC GmbH (Ammerbuch, Germany) (see Figure S7). Principal component analysis (PCA) was carried out by using the multivariate data analysis software SIMCA (17.0.2) from Sartorius Stedim Data Analytics AB (Umeå, Sweden). Data visualization was carried out using OriginPro 2022 (OriginLab, Northampton, MA, USA). More detailed information is given in respective figure captions or subchapters.

### 2.3 Preparation of Modified Silica Particles

#### 2.3.1 Preparation of Thiol Silica by Functionalization with Silatrane

In the first step, 2 g bare silica particles (3 µm, 300 Å, 100 m^2^/g) were dispersed in 20 mL of MilliQ water and sonicated for 5 min within a glass flask. Thereafter, the suspension was transferred to a glass funnel of porosity 5, and the water was removed by applying a slight vacuum for 16 h. Afterward, 3.5 g of the humidified silica was dispersed in 16 mL toluene and 4 mL methanol within a triple neck flask equipped with a mechanical stirrer, a nitrogen supply, and a reflux condenser. Then, 3‐meracptopropylsilatrane (8 µmol/m^2^) and DMAP (0.4 µmol/m^2^), both referring to dry silica, were added to the suspension, and the mixture was heated to reflux for 7 h. In the next step, the silica was washed three times with boiling methanol and toluene, each using a glass funnel with porosity 5. Thereafter, the modified silica particles were dried in a vacuum chamber at 60°C for 12 h. The amount of adsorbed water on the silica surface at the beginning of the grafting procedure was determined in accordance with the European Pharmacopeia. Thus, the humidified silica was dried in a heating chamber at 110°C for 96 h, and the drying loss was determined by weighing the silica before and after the drying process. Elemental analysis results for the modified silica can be found in Table 1.

**TABLE 1:** Elemental analysis results and calculated ligand densities.

SP	C^a^ [w‐%]	H^a^ [w‐%]	N^a^ [w‐%]	S^a^ [w‐%]	C^b^ [µmol/m^2^]	Residual SH‐ligand^b^ [µmol/m^2^]	Residual SH‐ligand^c^ [µmol/m^2^]	C18‐ligand^d^ [µmol/m^2^]	C4‐ligand^d^ [µmol/m^2^]	Triphenyl‐ligand^e^ [µmol/m^2^]	SO_3_H‐ligand^f^ [µmol/m^2^]
C4‐SP	1.46 ± 0.04	0.45 ± 0.01	< 0.03	< 0.02	9.72	—		—	2.43	—	—
C18‐SP	2.74 ± 0.05	0.64 ± 0.01	< 0.03	< 0.02	22.81	—		1.20	—	—	—
SH‐SP	1.88 ± 0.01	0.43 ± 0.00	< 0.03	1.32 ± 0.02	15.65	4.12	3.79	—	—	—	—
Triphenyl‐SP	6.82 ± 0.02	0.88 ± 0.01	< 0.03	1.29 ± 0.02	56.78	2.06	1.74	—	—	1.96	—
Triphenyl‐SAX‐SP	4.22 ± 0.01	0.68 ± 0.01	< 0.03	1.24 ± 0.00	35.13	2.01	1.49	—	—	0.93	—
Triphenyl‐ZWIX‐SP	3.94 ± 0.04	0.65 ± 0.01	< 0.03	1.20 ± 0.02	32.80	—	< 0.1	—	—	0.82	2.10

^a^ Determined by elemental analysis.

^b^ Calculated based on sulfur content (EA) and quantity of attached ligand assuming complete reaction (note, surface area of non‐modified silica was used for all calculations of surface coverages).

^c^ Determined by DPDS assay for determination of reactive sulfhydryls.

^d^ Calculated based in elemental analysis data assuming bifunctional bonding.

^e^ Calculated based on elemental analysis data of SH‐SP and the respective SPs.

^f^ Calculated assuming full oxidation.

#### 2.3.2 Preparation of Triphenyl‐Stationary Phase by Thiol‐Ene Click Reaction

Around 0.5 g dry thiol‐modified silica particles (SH‐SP) were suspended in 20 mL anhydrous toluene within a triple neck flask equipped with a mechanical stirrer, a reflux condenser, and a nitrogen supply. After adding allyltriphenylsilane (6 µmol/m^2^) and AIBN (5 mol% related to the quantity of ene‐groups) to the suspension, the reaction mixture was heated up to reflux, and the reaction was allowed to proceed for 7 h under continuous stirring and nitrogen rinsing. Thereafter, the modified silica particles were washed with boiling toluene and boiling methanol three times each using a glass funnel of porosity 5 and subsequently dried in a vacuum oven at 60°C for 24 h. Elemental analysis results for the modified silica can be found in Table 1.

#### 2.3.3 Preparation of Triphenyl‐SAX Stationary Phase by Thiol‐Yne Click Reaction

One gram dried thiol‐modified silica particles were suspended in 20 mL anhydrous methanol within a triple neck flask equipped with a mechanical stirrer, a reflux condenser, and a nitrogen supply. After adding 2‐propinyl‐triphenylphosphonium bromide (6 µmol/m^2^) and AIBN (10 mol% related to the quantity of yne‐groups) to the suspension, the reaction mixture was heated up to reflux, and the reaction was allowed to proceed for 7 h under continuous stirring and nitrogen rinsing. Thereafter, the modified silica particles were washed with boiling methanol and boiling toluene three times each using a glass funnel of porosity 5 and subsequently dried in a vacuum oven at 60°C for 24 h. Elemental analysis results for the modified silica can be found in Table 1.

#### 2.3.4 Preparation of Triphenyl‐ZWIX Stationary Phase by Oxidation of Triphenyl‐SAX

Around 0.5 g triphenylphosphonium‐modified silica particles were dispersed in a mixture of 40 mL methanol and 2.1 mL formic acid. Subsequently, a mixture of 0.5 mL hydrogen peroxide (30%, v/v) and 9.5 mL formic acid was added dropwise to the suspension. Thereafter, the reaction was allowed to proceed for 4 h under ice bath cooling and permanent mechanical stirring. Finally, the silica was washed three times with boiling methanol using a glass funnel of porosity 5 and dried in a vacuum oven at 60°C for 24 h. Elemental analysis results for the modified silica can be found in Table 1.

## 3 Results and Discussion

### 3.1 Trifunctional Bonded Platform Thiol‐Silica by Silatrane Immobilization

**FIGURE 1:**
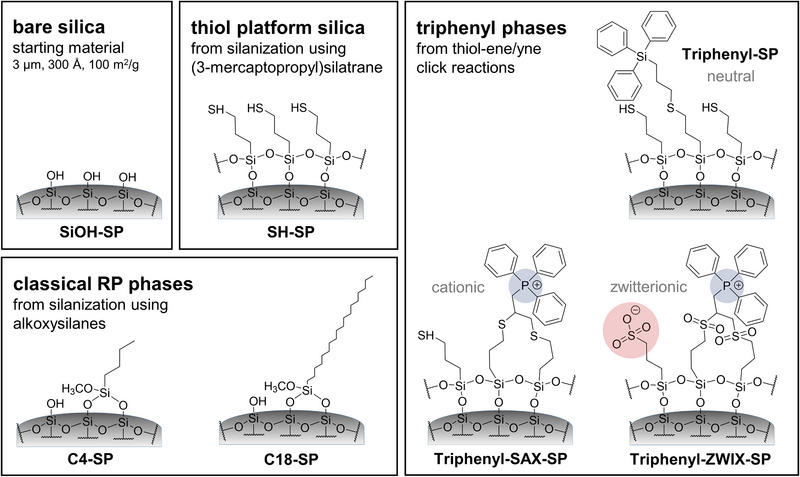
Surface chemistries of the prepared stationary phases. Synthesis procedures for C4‐SP and C18‐SP are described in the supplementary material (see Figure S3). All stationary phases were manufactured starting from the same spherical silica (3 µm, 300 Å, 100 m^2^/g).

**FIGURE 2:**
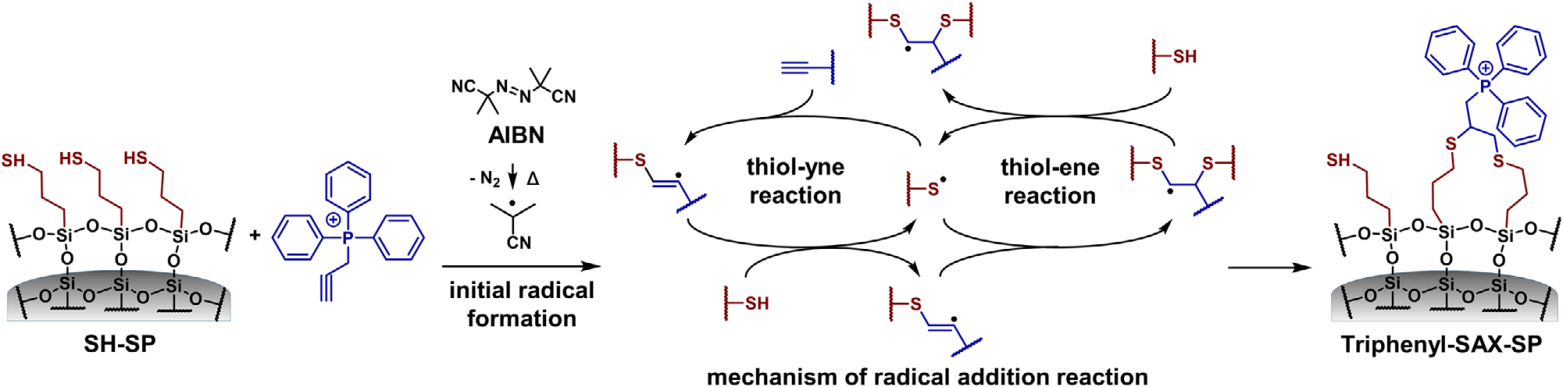
Synthesis scheme for the preparation of the triphenyl‐SAX‐SP by thiol‐yne/ene click reaction. After thermally initiated radical formation, the radical is transferred from the decomposition product of the initiator to the thiol groups. The thiyl radical generated causes a hydrothiolation reaction on the double or triple bonds of the reaction partners. As a result, the formation of a thioether takes place subsequently. In this process, a carbon‐centered radical is formed intermediately, which provokes a prolonged reaction by transferring the radical to the next thiol group. Therefore, double bonds can react once with one thiol group, whereas triple bonds can react with up to two thiol groups.

**FIGURE 3:**
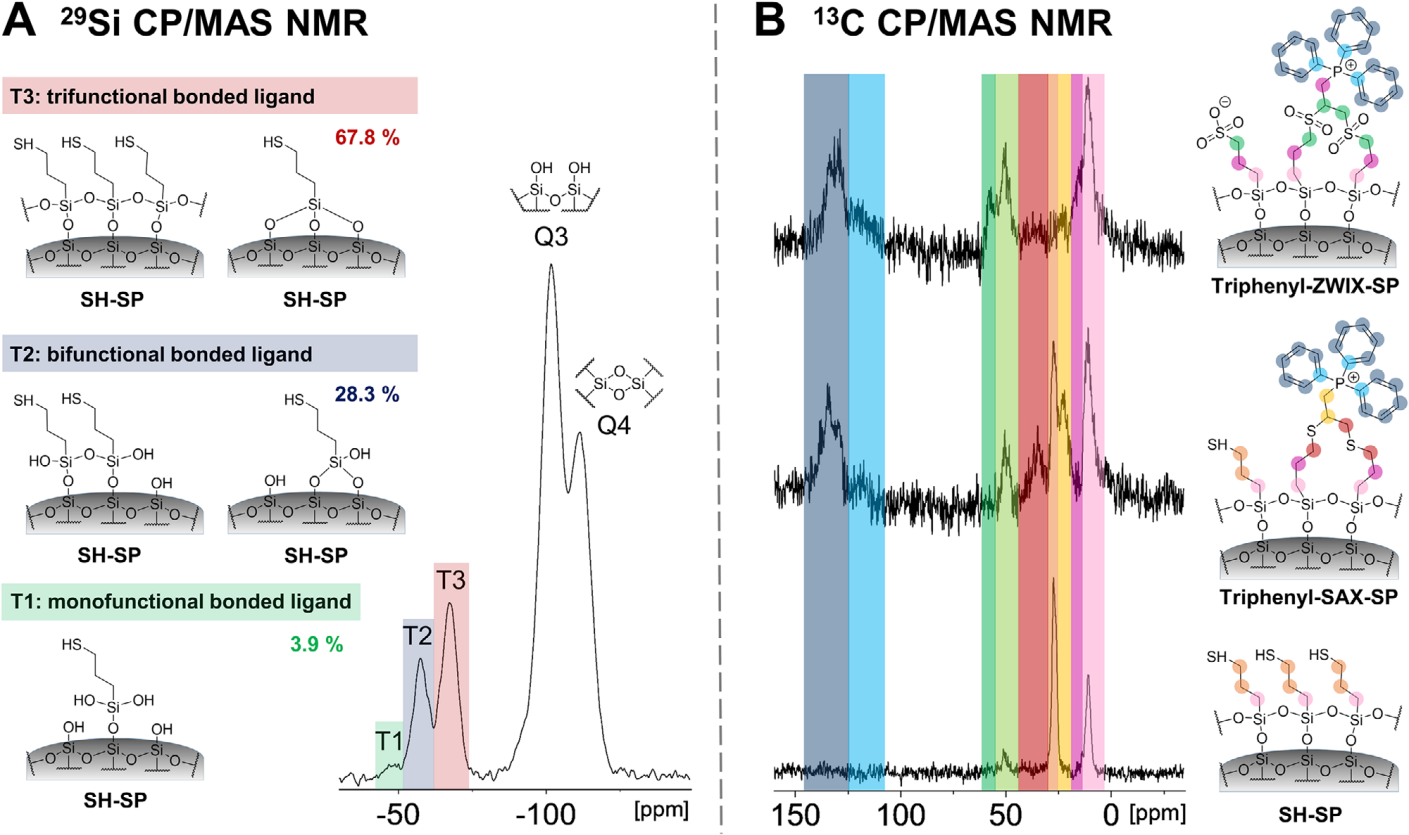
(A) ^29^Si CP/MAS NMR analysis of SH‐SP and (B) ^13^C CP/MAS NMR spectra for SH‐SP, triphenyl‐SAX‐SP and triphenyl‐ZWIX‐SP. (A) The ^29^Si NMR spectrum of SH‐SP exhibits the three characteristic signals for modified silica (T1, T2, and T3) beside the typical signals for the silica support (Q3: free single and vicinal silanol groups, Q4: siloxane groups). T1, T2, and T3 can be assigned to the silicon atoms of the diverse 3‐mercaptopropyl siloxane bonds immobilized on the silica surface, giving information about the proportion of mono‐, di‐, and trifunctional siloxane‐linked moieties. (B) ^13^C NMR spectra illustrate the successful synthesis of SH‐SP, triphenyl‐SAX‐SP, and triphenyl‐ZWIX‐SP. Thus, the successful immobilization of the phenyl ligands is proven as well as the accomplished oxidation of the residual sulfhydryl groups. The signal at approximately 50 ppm (bright green) can be attributed to methoxy groups generated on the silica surface due to the methanolic solvent used in the synthesis.

Thiolated brush‐type silica (Figure 1) is a frequently used carrier for the preparation of further functionalized silica by efficient and straightforward thiol‐ene or thiol‐yne click reactions (Figure 2). Usually, such thiol silicas are prepared by classical silylation reactions through condensation using functional trialkoxysilanes. Such a reaction leads mainly to immobilization by bifunctional siloxane bonding (vide infra). C4‐SP and C18‐SP, which were synthesized for comparison, were prepared conventionally utilizing the respective trialkoxysilanes (see Figure S3). In this study, (3‐mercaptopropyl)silatrane was employed for the preparation of the triphenyl phases (see Figure S4). The beneficial properties of silatranes over traditionally used alkoxysilanes for the bonding of chromatographic ligands to the silica surface were recently demonstrated [28]. Due to their cage‐like structure and the transannular N→Si bond, silatranes are not prone to hydrolysis under aqueous conditions and thus do not form oligomeric/polymeric structures in the reaction solution. However, silatranes can undergo condensation reactions with the silanols located on the silica surface due to acid catalysis of silica (Figure S5) [31, 32, 33]. Consequently, the formation of thin self‐assembling sulfhydryl layers takes place on the surface. In the previous study (see [28]), a two‐step synthesis approach was necessary to form the favorable self‐assembled polysiloxane surface layer. Under nonaqueous conditions, in the first step, the triethanolamine moiety remains attached to the silica surface (see Figure S5). It needs to be removed by a second hydrolysis step. In order to simplify the synthesis and reduce overall reaction time, a single‐step synthesis approach was developed in this study. The key to making a single‐step silanization with silatranes possible is the humidification of the silica prior to the silanization reaction to ensure that all triethanolamine moieties are cleaved off in the course of the reaction. Herein, the water content of the humidified silica was determined to be 48% (w/w). Subsequently, the resultant thiol silica was analyzed by elemental analysis (see Table 1) and solid‐state ^13^C and ^29^Si CP/MAS NMR spectroscopy (see Figure 3). It showed similar results in agreement with the two‐step silanization approach using silatrane in the previous study [28]. It can be seen from Table 1 that a very high density of incorporated sulfhydryl groups (4.12 µmol/m^2^) was obtained by the single‐step silanization reaction with silatrane. It indicates a polysiloxane layer on the silica surface rather than a trifunctional brush‐type immobilization, which does not reach such high surface coverage (see Figure S6). The ^29^Si CP/MAS NMR spectrum (Figure 3A) reveals that the thiol ligands are mainly attached by trifunctional siloxane bonding to the support (T3: 67.8%) with less bifunctional bonding (T2: 28.3%). There are very few monofunctional siloxane‐bonded ligands (T1: 3.9%). In contrast to the findings of the previous two‐step study, however, more than 90% of the thiol groups calculated by elemental analysis could also be detected by means of a thiol‐disulfide exchange reaction, indicating the accessibility and reactivity of the sulfhydryl moieties incorporated by this single‐step approach. It can be concluded that the present single‐step surface functionalization leads to less oxidation than the previous two‐step approach. It is favorable as a higher concentration of reactive surface sulfhydryls is available for ligand attachment. These findings indicate the superiority of the new single‐step grafting approach and make it a viable way to overcome the accessibility problems of the sulfhydryl groups that occurred by earlier synthesis approaches.

### 3.2 Immobilization of Triphenyl Ligands by Thiol‐Yne/Ene Click Reaction

A set of diverse triphenyl‐modified stationary phases was prepared by further decoration of the thiol platform silica by efficient immobilization through thiol‐ene/yne click reactions. These radical addition reactions are simple, generally highly efficient, proceed with high yields, and are characterized by fast reaction kinetics, high selectivities, and mild reaction conditions. They are therefore a powerful chemical strategy for surface modification of silica involving the reaction between a thiol and an ene‐ or yne‐group‐bearing component. As a result, the formation of one or two new carbon‐sulfur bonds occurs, respectively [29, 34, 35]. For the preparation of uncharged triphenyl‐SP, allyltriphenylsilane was used as the ene component, and thermal initiation with AIBN as radical initiator was applied (see Figure S4). Elemental analysis results revealed that about 50% of the determined sulfhydryl groups reacted with the triphenyl‐ligand providing a calculated ligand coverage of 1.96 µmol/m^2^ for the triphenyl‐SP (see Table 1). This was significantly higher compared with the quantity of immobilized *tert*‐butyl quinine (0.25 µmol/m^2^) reported in the previous study (see [28]) for the thiol‐ene reaction on thiol‐silica from a two‐step synthesis approach. This stationary phase still contained around 1.74 µmol/m^2^ reactive sulfhydryls after ligand immobilization.

The permanently positively charged triphenyl‐SAX‐SP was prepared with 2‐propinyl‐triphenylphosphonium bromide as the yne component (see Figure S4). The reaction mechanism is schematically illustrated in Figure 2. While ene components can only react with a single sulfhydryl group on the silica surface, leading to the anti‐Markovnikov product, yne components can react with two sulfhydryl moieties (Figure 2). This might be beneficial since it leads to doubly tethered linkages to the surface resulting in higher chemical stability of the surface chemistry and lower ligand bleeding, respectively. From elemental analysis data, a surface coverage of 0.93 µmol/m^2^ was calculated for the triphenyl‐SAX‐SP (see Table 1). A relative ligand density ratio between triphenyl‐SP (thiol‐ene, 1 thiol per ene) and triphenyl‐SAX‐SP (thiol‐yne, two thiols per yne) of 2.1:1.0 was calculated from EA, which corresponds almost exactly to the ratio of reactions with thiol groups possible per immobilized ligand (2:1, yne‐/ene‐component). The ^13^C CP/MAS NMR spectrum of the triphenyl‐SAX‐SP shows additional signals from the thioether‐bonded alkyne group between 30 and 40 ppm and the phenyl residues at around 120–140 ppm confirming the successful immobilization of the ligand (see Figure 3). In contrast to the triphenyl‐SP, the triphenyl‐SAX‐SP offers an additional anion exchange site due to the central phosphonium group, which is surrounded by three bulky phenyl groups. Such bulky moieties were assumed to shield the charged site from short‐range ionic interactions with counterionic analytes and diminish silanol interactions of the analytes by electrostatic shielding or steric hindrance.

### 3.3 Generation of Zwitterionic Surface by Oxidation of Residual Thiols

Zwitterionic surfaces have shown interesting and promising properties in LC, in particular in HILIC and ion chromatography. In order to insert additional strong cation‐exchange sites and enable attractive and repulsive electrostatic interactions simultaneously, triphenyl‐SAX‐SP was oxidized using performic acid. Performic acid is an efficient oxidizing agent that can be used under mild conditions at which silica and siloxane bonds are stable. Hereby, unreacted residual sulfhydryl groups were oxidized to sulfonic acid moieties, and consequently a zwitterionic surface was created [36]. Contrary to classical sulfobetaine phases widely used in HILIC, opposite charges were on distinct ligands and farther apart from each other, probably leading to imperfect charge compensation.

**FIGURE 4:**
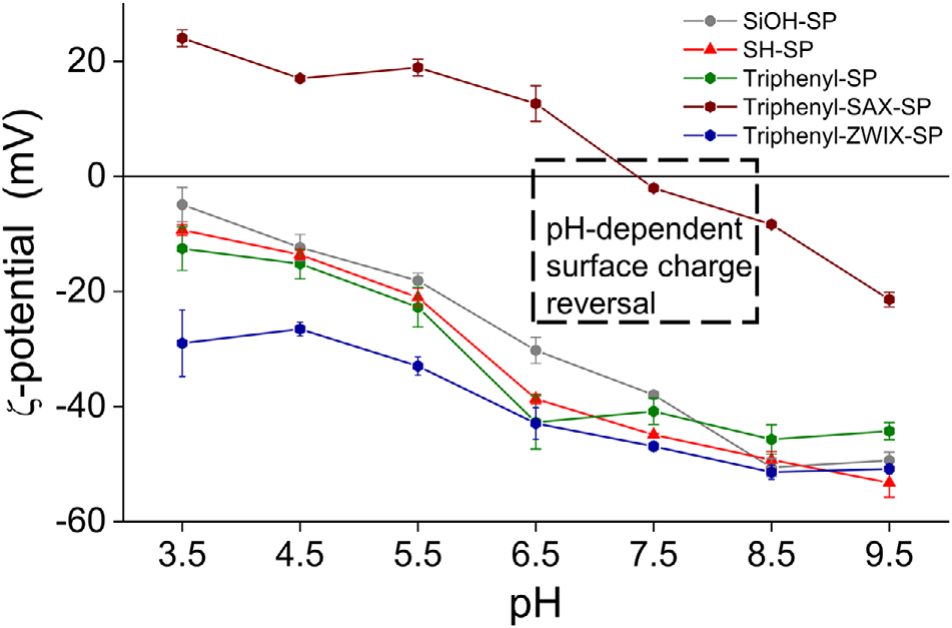
ζ Potentials of the modified silica particles determined by electrophoretic light scattering at different pH values (10 mM KCl in 1 mM buffer).

The successful oxidation was proven by determination of sulfhydryl groups via a thiol‐disulfide exchange reaction using DPDS, ^13^C CP/MAS NMR analysis, and pH‐dependent ζ‐potential measurements (vide infra). After the oxidation procedure, no remaining residual thiol groups could be detected by the DPDS assay (see Table 1). According to elemental analysis, there is a 2‐fold excess of sulfonic acid moieties compared to phosphonium groups, which may lead to the assumption that the surface is net negatively charged (see ζ‐potential measurements in Figure 4). In the ^13^C NMR spectrum, typical signals for the mercaptopropyl moiety at around 20–30 ppm disappeared or were shifted, being indicative of a successful oxidation of both sulfhydryls and thioethers of triphenyl‐SAX‐SP (see Figure 3). Sulfhydryls are oxidized to sulfonic acids while thioethers get oxidized to sulfone groups.

### 3.4 Surface Charge Characterization by ζ‐Potential Measurements

ζ‐Potentials have been determined by electrophoretic light scattering with suspensions of the (modified) silica particles in a solution of 10 mM KCl in 1 mM buffer [37]. It allows one to indicate changes in the surface chemistry and gives an orientation of the charge state under distinct chromatographic conditions. Thus, ζ‐potentials were recorded for all materials, including the bare silica support (SiOH‐SP), at different pH values in the range between pH 3.5 and 9.5. It can be seen in Figure 4 that bare silica shows negative ζ‐potentials over the entire pH range. With increasing pH, the surface charge increases as more and more silanols get dissociated, and hence the negative ζ‐potentials increase from −5 mV at pH 3.5 (silanols mostly non‐dissociated) to −50 mV (silanols fully dissociated). There is a slight change in the ζ‐potentials of the thiol‐modified particles and the triphenyl‐SP with a neutral surface bonding structure to more negative values, which appears unexpected at first instance as some silanols are modified and hence less residual silanols are expected to be available. However, the aqueous treatment of the bare silica prior to thiol modification (prewetting step) can lead to the hydrolytic cleavage of siloxane bonds and consequently to the formation of new silanol groups. Thus, the net charge of SH‐SP and triphenyl‐SP was found to be more negative than for precursor SiOH‐SP. In sharp contrast, modification of the SH‐SP particles with triphenylphosphonium moieties in triphenyl‐SAX‐SP shifted its ζ‐potential to positive values in the low pH range, being indicative of a net positive surface charge. However, charge reversal (umpolung) occurs for this material at approximately pH 7.5, leading to negative surface charges above this pH value due to the increasing deprotonation of the silanols. Furthermore, ζ‐potentials of the triphenyl‐ZWIX‐SP particles dropped significantly after oxidation by −30 mV (high pH) to −50 mV (low pH) due to the embedded, permanently negatively charged sulfonate groups which confirms the successful oxidation of the residual thiols as derived from the DPDS assay and elemental analysis. It also indicates the excess of sulfonate groups, over phosphonium moieties (see Figure 4).

### 3.5 Chromatographic Evaluation

**FIGURE 5:**
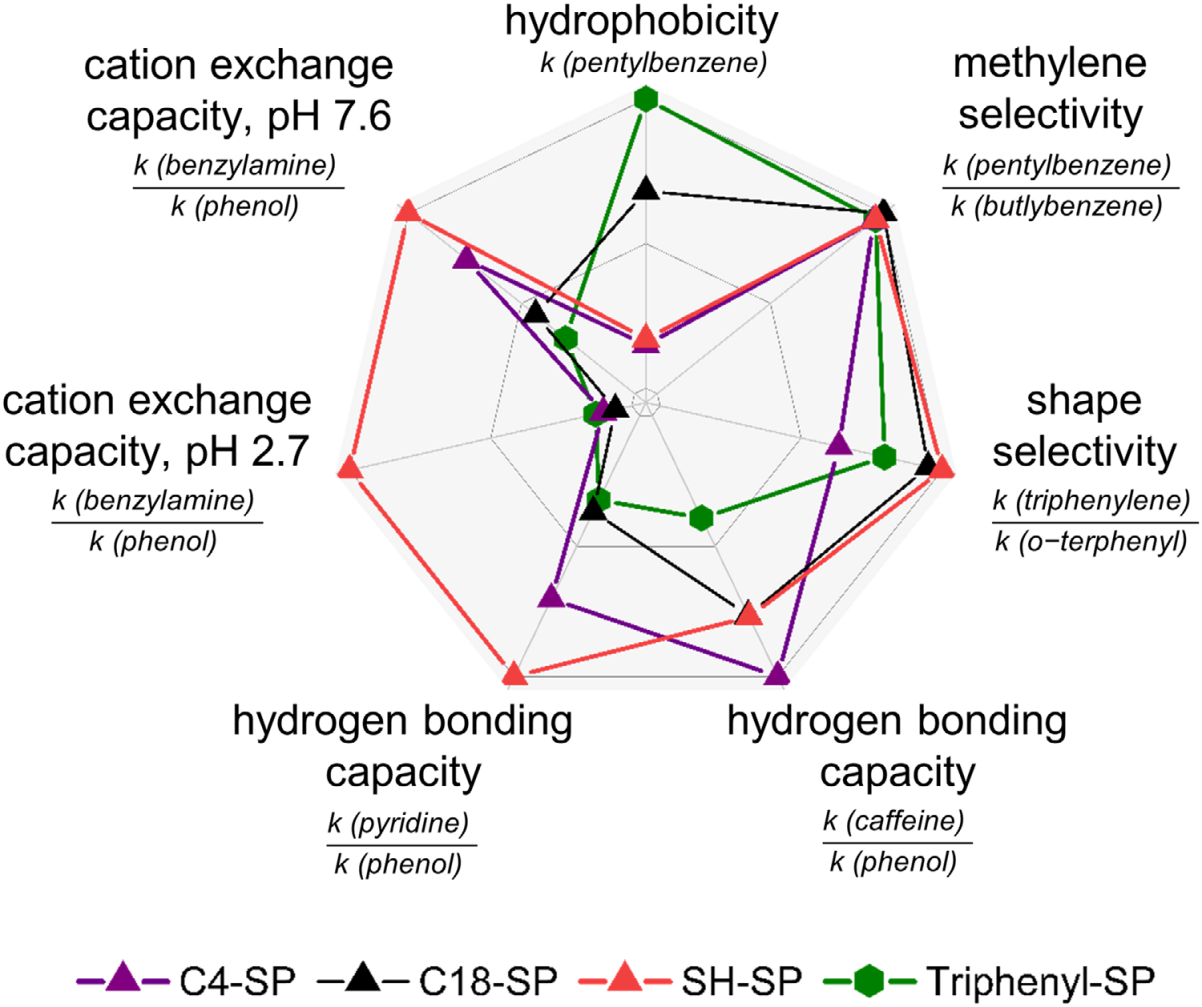
Normalized radar plot based on chromatographic investigation under Tanaka test conditions for RP‐type phases. Further information concerning chromatographic conditions and parameters can be found in Figures S8 and S9 and Tables S1 and S2.

As the first chromatographic test to characterize primarily the RP‐type triphenyl‐SP with neutral surface bonding in comparison to C4‐ and C18‐SP as benchmarks, a Tanaka test was performed on all columns [38, 39, 40]. The different properties tested and analytes used as probes to do so, as well as corresponding calculations of characteristic values (typically obtained as separation factors of the ratio of the retention factors of two specific analytes), are briefly summarized in Table S1. The results of the Tanaka test achieved for the various SPs are depicted in suppl. Table S2, and a graphical representation by a spider plot is given in Figure 5. The Tanaka test is designed to compare RP‐type phases, and therefore the results are particularly meaningful for RP‐type phases. The results for ionic mixed‐mode phases (see Figure S8) must be interpreted with caution, taking into account the effects of ion‐exchange sites, which are not present in classical RP‐type phases (with the exception of pH‐dependent ionized residual silanols).

Since all stationary phases were based on 300 Å pore size silica, the hydrophobicity of the SPs and hydrophobic retention (as measured by *k* of pentylbenzene), respectively, are relatively small as compared to standard 100 Å RP18 stationary phases. It declined in the order of triphenyl‐SP > C18‐SP > C4 ∼ SH‐SP, roughly in the order of the carbon content determined by elemental analysis (see Figure 5) (note, mixed‐mode phases will be discussed separately below). Accordingly, also the hydrophobic selectivity was smaller herein than on 100 Å RP18 stationary phases (C18‐SP > triphenyl‐SP ∼ SH‐SP > C4‐SP). The shape selectivity was the highest for the SH‐SP, as the 3‐mercaptopropyl ligands were most densely packed, followed by the C18‐SP > triphenyl‐SP > C4‐SP (Figure 5). The separation factors between caffeine and phenol, as well as pyridine and phenol, represent the hydrogen acceptor and hydrogen donor capacities of the SPs, respectively (collectively hydrogen bonding capacities). The radar plot in Figure 5 reveals that the triphenyl‐SP exhibits the lowest hydrogen bonding capacities of these four RP‐type phases, both regarding H‐acceptor and H‐donor capacities. Since it depends on the available silanol moieties, it can be concluded that the triphenyl‐phase has fewer silanol interactions than the other three RP‐type phases tested. In contrast to C4‐ and C18‐SP, the triphenyl‐SP exhibits therefore also less cation exchange properties at pH 2.6 and pH 7.6, which indicates a better shielding of the analytes from the surface silanols as well. Furthermore, the high value for silanol activity of the precursor SH‐SP may be partly explained by the weakly acidic sulfhydryls, which may contribute to this parameter.

Table S2 and Figure S8 also show the corresponding results of the mixed‐mode ion‐exchange phases triphenyl‐SAX‐SP and triphenyl‐ZWIX‐SP (for structures of the analytes employed for the Tanaka test, see Figure S9). Both mixed‐mode ion‐exchange phases have lower hydrophobicity due to the charged phosphonium moiety and sulfonic acid residues, respectively, and lose their methylene selectivity. On the other hand, both mixed‐mode ion‐exchange phases show surprisingly high shape selectivity, which is significantly higher than the one of triphenyl‐SP (Table S2). It is striking that this is an incident with the type of immobilization by thiol‐yne click reaction, which leads to doubly tethered chromatographic ligands imposing rigidity to the pendant triphenyl phosphonium selector. The H‐bond acceptor properties of the triphenyl‐SAX‐SP are comparable to those of the triphenyl‐SP (since it depends on the residual silanol surface, which is similar in the two phases). On the triphenyl‐ZWIX‐SP, the H‐acceptor properties are significantly enhanced due to sulfone and sulfonate moieties. The test for the H‐donor properties (*k*_pyridine_/*k*_phenol_) of the triphenyl‐SAX‐SP is affected by superimposed repulsive electrostatic interactions between the phosphonium moiety and the pyridine probe, perturbing the assessment of the H‐donor properties of the SP. On the contrary, on triphenyl‐ZWIX‐SP, a cation‐exchange process is superimposed so that the H‐donor test is actually additively composed of the sum of retention increments from H‐donor interactions and cation‐exchange. The cation exchange test at pH 2.7 shows strong repulsive interactions between the phosphonium moiety and benzylammonium probe of the Tanaka test on the triphenyl‐SAX‐SP, while a strong cation‐exchange retention mechanism is imposed on the triphenyl‐ZWIX‐SP due to sulfonic acid moieties. At pH 7.6, the triphenyl‐SAX‐SP is close to its pI (near the neutral net surface), and hence repulsive electrostatic interactions as well as, cation‐exchange (with dissociated silanols), become negligible. In contrast, on the triphenyl‐ZWIX‐SP a strong cation exchange process gets activated at pH 7.6 for benzylammonium as expected from the sulfonic acid groups.

**FIGURE 6:**
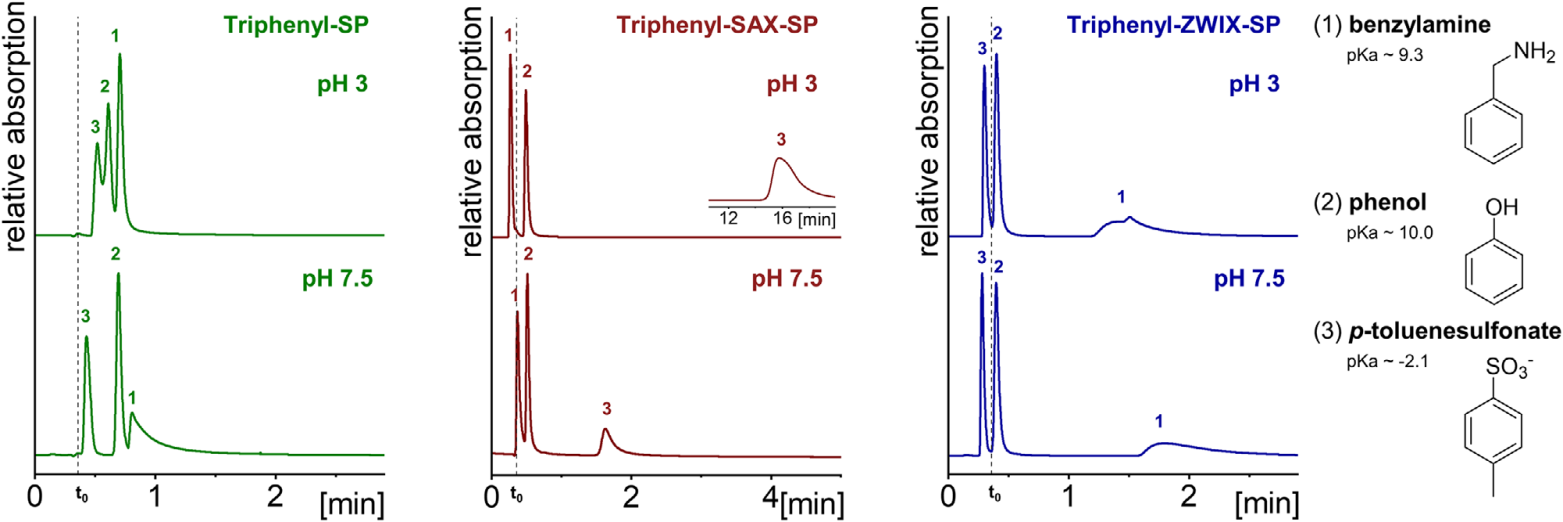
Evaluation of the ion exchange characteristics of the triphenyl‐SP, triphenyl‐SAX‐SP and triphenyl‐ZWIX‐SP at pH 3 and pH 7.5. Chromatographic conditions: The mobile phase consisted of MeOH/aqueous ammonium phosphate buffer (20 mM, adjusted to pH 3 or pH 7.5) (30/70, v/v), flow rate: 1 mL/min, temperature: 25°C, injection volume: 5 µL.

In addition, the surface charge characteristics were investigated chromatographically by another test mixture consisting of a set of three different analytes with varying pK_a_ values (acidic, neutral, basic) that were applied at different mobile phase pH values (pH 3 and 7.5). The results are illustrated in Figure 6 and Table S3. Here, both triphenyl‐SP (due to residual silanols) and triphenyl‐ZWIX‐SP (due to sulfonic acid moieties) demonstrated cation‐exchange characteristics at both pH values. Thus, benzylamine eluted last on both columns. The negatively charged analyte *p*‐toluenesulfonate eluted first on these two SPs due to repulsive electrostatic interactions and interestingly prior to the dead volume marker on the triphenyl‐ZWIX‐SP due to (partial) ion‐exclusion from the pores. In contrast, there was still retention observed for the analyte on triphenyl‐SP, most probably due to the higher proportion of hydrophobic interactions (cf. ligand densities; Table 1) and less accessible anionic interaction sites. The triphenyl‐SAX‐SP, on the other hand, showed a different behavior and exhibited anion‐exchange properties, as expected. Consequently, the elution order was reversed in comparison to the two other triphenyl‐modified SPs, and the anionic analyte eluted last. However, its retention was dramatically reduced by switching the pH from 3 to 7.5, which is quite common for silica‐based anion exchangers, indicating the immense influence of the deprotonated silanols on the surface charge. According to the determined ζ‐potentials, even a reversal of the elution order could be expected above pH 7.5. In this case, however, the net charge does probably not correspond exactly to the chromatographically accessible charged interaction sites at the surface, as the bulky triphenyl groups might impede the penetration of the analytes to the unmodified silica surface and therefore suppress direct interactions with the deprotonated silanol groups. Instead, the benzylammonium analyte is repelled from the surface by the phosphonium moiety and elutes therefore before *t*_0_ with a slight ion‐exclusion effect.

### 3.6 Stationary Phase Classification via PCA

**FIGURE 7:**
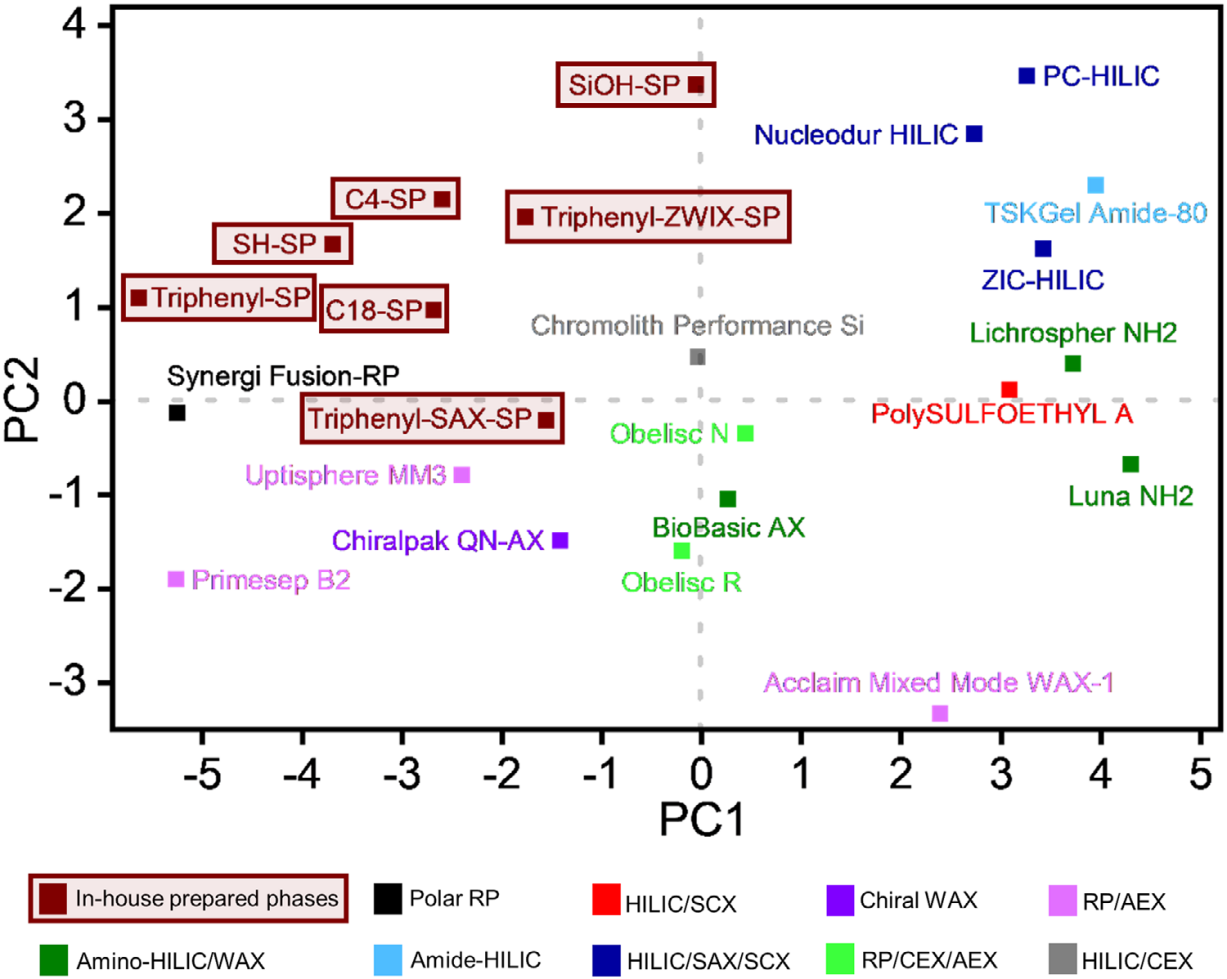
Score plot of principal component analysis (PCA) for column classification. The corresponding loading plot can be found in Figure S13.

In order to classify the novel stationary phases within a set of commercially available columns, standard HILIC and RPLC tests were performed, and the resultant retention factors subjected to PCA. Chromatographic conditions, data obtained, and the surface chemistries of commercial columns are illustrated and summarized in Figures S10–S13 and Table S4. The score plot is depicted in Figure 7. Here, apparently, the latent variable PC1 encodes mostly for the hydrophobicity of the stationary phases (which decreases from low to high PC1), whereas PC2 reflects primarily the surface charge (net charge positive at low PC2 values and negative at high PC2).

As expected, amongst the new triphenyl phases, triphenyl‐SP was found to be the phase with the most pronounced hydrophobic properties, which is underpinned by its close clustering to Synergi Fusion‐RP (which is a polar embedded C18 phase that offers balanced polar and hydrophobic selectivity). This hydrophobic character was reduced by the introduction of the ionic interaction sites in triphenyl‐SAX‐SP and triphenyl‐ZWIX‐SP, which are located on the PC1 scale between the classic alkyl phases (C4‐ and C8‐SP), which are more hydrophobic, and pure silica (SiOH‐SP), which is less hydrophobic. In terms of hydrophobicities, these two mixed‐mode phases are similar to those of Uptisphere MM3 (RP phase with cationic endcapping; octyl/strong anion exchanger) and Chiralpak QN‐AX (chiral stationary phase with properties resembling achiral RP/WAX columns). The zwitterionic mixed‐mode phase Obelisc R is a bit less hydrophobic. In terms of surface charge, triphenyl‐SP is located closely to C18‐SP, which illustrates the shielding effect of the bulky phenyl moieties similar to the long octadecyl moieties. Due to the shielding, silanols are less accessible for chromatographic interactions with the analytes than it is the case for phases containing smaller ligands, which cannot shield the silanols (cf. C4‐SP). On the contrary, triphenyl‐SAX‐SP and triphenyl‐ZWIX‐SP exhibited more distinctive surface charge characteristics. Thus, triphenyl‐SAX‐SP showed a slightly positive surface charge, according to the PCA score plot, similar to the zwitterionic mixed‐mode phase Obelisc N with a terminal positively charged group and an interior negatively charged functional group embedded in the alkyl ligand strand (see Figure S12) and the amino phase Uptisphere MM3 (octyl‐SP with SAX endcapping). The zwitterionic mixed‐mode phase triphenyl‐ZWIX‐SP, on the other hand, is located between the two sulfobetaine‐type zwitterionic columns ZIC‐HILIC and Nucleodur HILIC in terms of PC2 (indicative of its negative surface charge). In conclusion, the three prepared phenyl‐phases cover a broad range of hydrophobicity and surface charge, leading to orthogonality of retention characteristics. This might make the set of triphenyl‐modified SPs a useful tool for a variety of analytical separations.

### 3.7 Analysis of Glycopeptide Teicoplanin Utilizing Triphenyl‐Modified Stationary Phases

**FIGURE 8:**
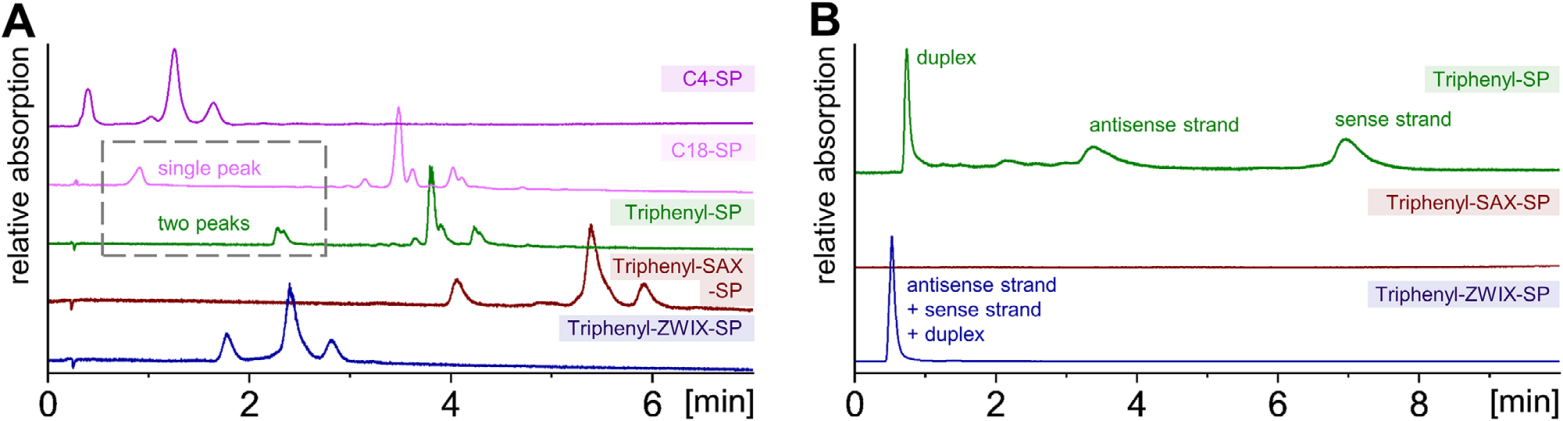
(A) Analysis of the biomolecules teicoplanin and (B) patisiran. The chemical structure of the biomolecules can be found in Figures S14 and S15. (A) Analysis of teicoplanin subspecies utilizing the set of triphenyl‐modified SPs and comparative RP‐SPs. Chromatographic conditions: Mobile Phase A consisted of water containing 10 mM ammonium acetate (pH adjusted to pH 5.4), mobile Phase B consisted of mobile Phase A and acetonitrile (5/95, v/v), flow rate: 1.0 mL/min, gradient time: 20 min (10% B to 100% B) temperature: 30°C, injection volume: 3 µL, detection: 210 nm. (B) Comparison of patisiran sense and antisense strand analysis on the three triphenyl‐modified SPs. Chromatographic conditions: Mobile Phase A consisted of water containing 20 mM ammonium acetate, mobile Phase B consisted of mobile Phase A and methanol (90/10, v/v), flow rate: 0.6 mL/min, gradient time: 10 min (5% B to 20% B), temperature: 40°C, injection volume: 5 µL, detection: 254 nm.

Teicoplanin is a multicomponent antibiotic drug and consists of several glycopeptides varying in the attached fatty acid residue (see Figure S14). Thus, they differ mainly in hydrophobicity. Consequently, these different species are typically resolved by reversed‐phase chromatography [41, 42]. Accordingly, the two synthesized classical RP phases (C4‐SP and C18‐SP) were utilized as benchmarks for comparison. As it can be seen in Figure 8A, all columns were able to resolve at least three peaks. In this particular application, it seems that the SPs with neutral hydrophobic surface bonding (triphenyl‐SP and C18‐SP) are beneficial, as the glycopeptide variants differ in the lipophilicity of the fatty acyl side chains. The triphenyl‐SP shows stronger retention than the C18‐SP and some minor differences in the selectivity profile. The C4‐SP does not have enough hydrophobicity to sufficiently separate the different forms differing in fatty acyl hydrophobicity and branching. In contrast, the other two triphenyl mixed‐mode SPs offer additional electrostatic interactions. Obviously, these additional interactions caused peak broadening and an accompanying loss of resolution due to slow adsorption–desorption–kinetics of ion‐exchange processes. Since teicoplanin offers an excess of negatively charged sites due to the carboxylic acid function and the phenolic hydroxyl groups, repulsion on the net negatively charged triphenyl‐ZWIX‐SP leads to faster elution of the analytes than observed on the triphenyl‐SP. In contrast, the triphenyl‐SAX‐SP showed increased retention for the analytes due to the embedded positively charged phosphonium moieties leading to a superimposed anion exchange process. However, this is not of benefit for the present application, as the macro‐ and micro‐variants do not differ in charge nor hydrophilicity. Consequently, for this specific application, the triphenyl‐SP has a clear advantage over the triphenyl mixed‐mode SPs (triphenyl‐SAX‐SP, triphenyl‐ZWIX‐SP).

### 3.8 Analysis of Small Interfering Ribonucleic Acid Patisiran Utilizing Triphenyl‐Modified Stationary Phases

Patisiran is a small interfering ribonucleic acid (siRNA) that is utilized for the medical treatment of hereditary transthyretin‐mediated amyloidosis. It consists of two partially complementary single strands. The sense and the antisense strand are composed of 21 nucleotides that are linked via phosphodiester bonds; some of the 2′‐hydroxyls are methylated (indicated with m, see Figure S15). The two‐nucleotide overhang consists of desoxyribose residues. For liquid chromatography of oligonucleotides, ion‐pairing reversed‐phase liquid chromatography is usually the technique of choice. However, there is a quest for ion‐pair‐free RP or HILIC methods that prevent contamination of the LC‐MS system with ion‐pairing agents [43, 44, 45, 46, 47, 48].

In this study, ion‐pair reagent‐free reversed‐phase chromatography was carried out using 20 mM ammonium acetate buffer and a methanol gradient for elution. As it is shown in Figure 8B, the sense and antisense strands were well resolved on the triphenyl‐SP under these conditions. As can be seen from Figure S16, the selectivity between sense and antisense strands is significantly larger than in ion‐pair RPLC with BEH C18. It can be assumed that both hydrophobic and π─π‐interactions contribute to the separation of the two strands on the triphenyl‐SP. In this context, it should be mentioned that the separation was performed at a column temperature (40°C) close to the melting temperature of patisiran (*T*_m,calc _= 46.9°C). Hence, both duplex and single strands are visible. At temperatures significantly above *T*_m_, the duplex peak should disappear. The duplex species eluted earlier, close to *t*_0_, since the nucleobases are mutually saturated by intermolecular hydrogen bonding and base stacking and thus shielded from interaction with the triphenyl‐SP. Since there are multiple negatively charged phosphate groups freely available in the duplex siRNA, elution close to t_0_ might be driven by repulsive electrostatic interactions with dissociated silanols. In sharp contrast, ionic interactions play a decisive role in the retention and elution of patisiran on the other two triphenyl‐modified phases. Since the sugar‐phosphate backbones of the siRNA strands offer negatively charged interaction sites, strong retention occurred on the positively charged triphenyl‐SAX‐SP in accordance with an anion‐exchange process, and no elution was observed with typical RP conditions due to the multiply negatively charged analyte and a weak elution strength of the mobile phase (low ionic strength of 20 mM ammonium acetate). However, in anion‐exchange mode with gradient elution using 1 M NaCl, the patisiran single and duplex strands (injected as single component standards) could be eluted and were well separated (Figure S17A). In contrast, there was no retention observed on the zwitterionic triphenyl‐ZWIX‐SP, which possesses an overall negative net charge due to the excess of negatively charged sulfonate groups, and hence electrostatic repulsion leads to fast elution, even faster than the elution of the duplex species on triphenyl‐SP. These results suggest that a fine‐tuning of the mobile phase or of the proportion of negatively and positively charged sites on the triphenyl‐SPs can lead to a very useful stationary phase for charged analytes with combined adsorptive and repulsive interaction sites offering complementary selectivities.

## 4 Concluding Remarks

In this work, the preparation of a new set of triphenyl‐modified stationary phases, partially embedded with ion‐exchange properties leading to mixed‐mode RP/ion‐exchange phases, for high‐performance liquid chromatography was reported. For this purpose, thiol‐silica was utilized as a carrier and further decorated with triphenyl‐ligands by thiol‐ene/yne click immobilization reaction. Herein, also an innovative strategy utilizing silatrane chemistry for the synthesis of the thiol‐silica was applied. Thus, thiol‐silica with a great amount of trifunctional siloxane‐bonded ligands was obtained, and the accessibility of the sulfhydryl groups was preserved. Chromatographic tests indicated the increased shielding of silanols due to the introduction of a dense polysiloxane layer and bulky triphenyl moieties. The PCA illustrated the similarities and orthogonality to commercially available columns. Additionally, ζ‐potential determinations exhibited the differences in pH‐dependent surface charges of the prepared phases. Finally, the prepared columns were applied for liquid chromatography analysis of the biomolecules teicoplanin and patisiran. These analyses showed that there is still fine‐tuning of chromatographic conditions and charge properties of the phases needed. However, the concept seems to be promising, and further investigation will be part of future studies.

## Author Contributions

**Marc Wolter**: investigation, conceptualization, methodology, formal analysis, data curation, visualization, supervision, writing–original draft, writing–review and editing. **Mirna Maalouf**: investigation. **Mateusz Janek**: investigation. **Cornelius Knappe**: supervision. **Markus Kramer**: investigation. **Michael Lämmerhofer**: conceptualization, methodology, supervision, writing–review and editing, resources.

## Conflicts of Interest

The authors declare no conflicts of interest.

## Supporting information



Supporting Information

## Data Availability

The data that supports the findings of this study are available in the Supporting Information Material of this article.
