# Supplementary material for: Triphenyl‐Modified Mixed‐Mode Stationary Phases With and Without Embedded Ion‐Exchange Sites for High‐Performance Liquid Chromatography
Source: J Sep Sci. 2024 Dec 23;47(24):e70058. doi: 10.1002/jssc.70058 (PMC11667146; doi:10.1002/jssc.70058)
Supplement: Supplementary file 1 — Supporting Information [file JSSC-47-e70058-s001.docx]

**Supplementary Material**

**Triphenyl-modified mixed-mode stationary phases with and without embedded ion-exchange sites for high performance liquid chromatography**

Marc Wolter ^1^, Mirna Maalouf ^1^, Mateusz Janek ^1^, Cornelius Knappe ^1^, Markus Kramer ^2^, Michael Lämmerhofer ^1^

^1^ Institute of Pharmaceutical Sciences, Pharmaceutical (Bio-)Analysis, University of Tübingen, Auf der Morgenstelle 8, 72076 Tübingen, Germany

^2^ Institute of Organic Chemistry, University of Tübingen, Auf der Morgenstelle 18, 72076 Tübingen, Germany

Author for correspondence:

Prof. Dr. Michael Lämmerhofer

Pharmaceutical (Bio-)Analysis

Institute of Pharmaceutical Sciences

University of Tübingen

Auf der Morgenstelle 8

72076 Tübingen, Germany

T +49 7071 29 78793, F +49 7071 29 4565

E-mail: [michael.laemmerhofer@uni-tuebingen.de](mailto:michael.laemmerhofer@uni-tuebingen.de)

**Table of contents**

**PART I: Synthesis of (3-mercaptopropyl)silatrane**

▪ Figure S1 Reaction scheme for the synthesis of (3-mercaptopropyl)silatrane.

▪ Figure S2 NMR spectra of (3-mercaptopropyl)silatrane.

**PART II: Synthesis of modified silica particles and column packing**

▪ Figure S3 Reaction scheme for the preparation of C4-SP and C18-SP.

▪ Figure S4 Reaction scheme for the preparation of SH-SP, Triphenyl-SP,

Triphenyl-SAX-SP and Triphenyl-ZWIX-SP.

▪ Figure S5 Initial mechanism of silanol-catalyzed silica surface modification using (3-mercaptopropyl)silatrane.

▪ Figure S6 Surface chemistries of silatrane-modified silica.

▪ Figure S7 General scheme of the in-house performed column packing procedure.

**PART III: Chromatographic tests and stationary phase classification**

▪ Table S1 Chromatographic conditions of Tanaka test for RP phases.

▪ Figure S8 Normalized radar plots obtained from Tanaka test.

▪ Figure S9 Analytes applied in Tanaka test.

▪ Table S2 Results of Tanaka test.

▪ Table S3 Chromatographic data obtained from ion-exchange test.

▪ Figure S10 Chromatograms obtained from RP test and analyte applied.

▪ Figure S11 Analytes applied for the HILIC tests.

▪ Table S4 Retention factors obtained from RP and HILIC tests.

▪ Figure S12 Surface chemistries of commercial columns.

▪ Figure S13 Loadings scatter plot of principal component analysis: p1 vs. p2.

▪ Figure S14 Chemical structure of teicoplanin.

▪ Figure S15 Chemical structure of patisiran.

- Figure S16 Analysis of patisiran on Triphenyl-SP in RPLC mode and comparison with

ion-pair RPLC on BEH C18.

- Figure S17 Analysis of patisiran on Triphenyl-SAX-SP in anion-exchange mode and comparison with Triphenyl-ZWIX-SP (ion-exclusion mode).

**PART VI: References**

**PART I: Synthesis of (3-mercaptopropyl)silatrane**

**Fig. S1.** Synthesis of (3-mercaptopropyl)silatrane. The condensation reaction between (3-mercaptopropyl)trimethoxysilane and triethanolamine results in the formation of (3-mercaptopropyl)silatrane. Synthesis procedure: Initially, 0.1 mol (3-mercaptopropyl)trimethoxysilane and 0.1 mol triethanolamine were carefully weighed into a round bottom flask (250 mL). After adding 20 mL methanol and 2 mL methanolic sodium hydroxide solution (2 mg/mL) to the solution the flask was attached to a rotary evaporator and the solvent was slowly evaporated at 40 °C. Thereafter, the reaction was heated up to 60 °C and allowed to react for 12 h. Meanwhile, the methanol formed in the condensation reaction was steadily evaporated. Lastly, the obtained white product was recrystallized from hexane in order to give pure (3-mercaptopropyl)silatrane (MPS). The yield was 91 % and synthesis control was carried out by nuclear magnetic resonance (NMR) analysis revealing a product purity of 99 % according to ^1^H-NMR data. The corresponding NMR spectra are depicted in Fig. S2.


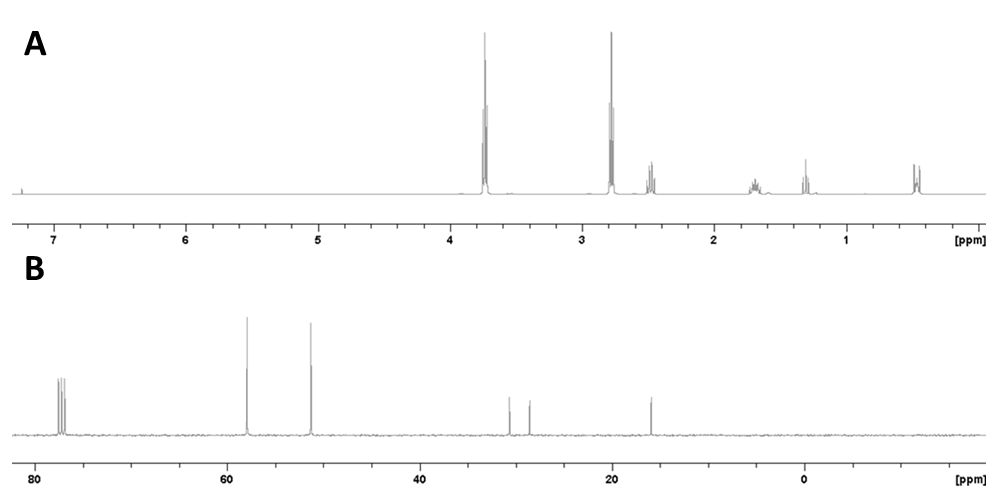


**Fig. S2.** NMR spectra of (3-mercaptopropyl)silatrane. A: ^1^H NMR spectrum ((J_t_ [Hz]), CDCl_3_, 400 MHz): δ 0.44-0.48 ppm (m, 2H), 1.31 ppm (t, Jt = 7.90 Hz, 1H), 1.69 ppm (quintet, Jt = 7.88 Hz, 2H), 2.78 ppm (t, Jt = 5.82 Hz, 6H) , 3.74 ppm (t, Jt = 5.82 Hz, 6H); B: ^13^C NMR (CDCl_3_, 100.6 MHz): δ 15.95 ppm (1 C), 28.58 ppm (1 C), 30.68 ppm (1 C), 51.32 ppm (3 C), 57.95 ppm (3 C). NMR spectra were calibrated to the solvent peaks with δ(1H) = 7.24 ppm and δ(13C) = 77.23 ppm.

**PART II: Synthesis of modified silica particles and column packing**


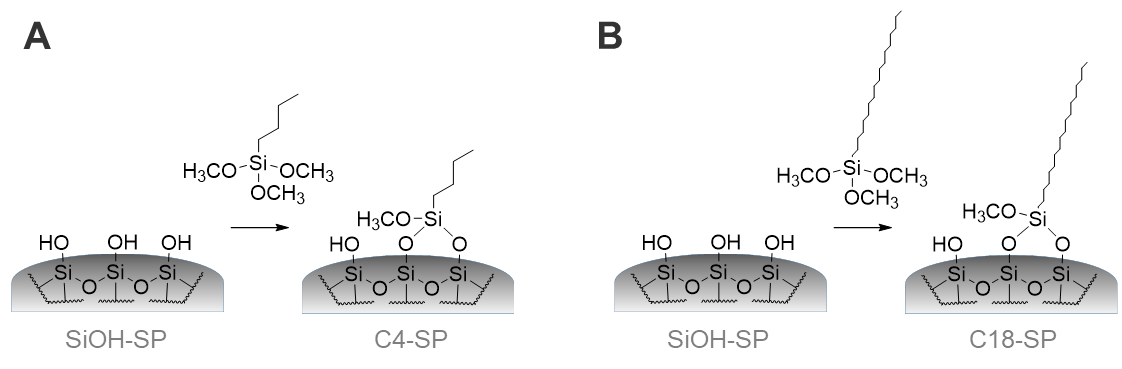


**Fig. S3.** Reaction scheme for the preparation of C4-SP (A) and C18-SP (B). Synthesis procedure: Initially, 0.5 g bare silica particles, n-butyltrimethoxysilane (6 µmol/m^2^) or n-octadecyltrimethoxysilane (6 µmol/m^2^), respectively, and DMAP (0.3 µmol/m^2^) were dispersed in 20 mL anhydrous toluene within a triple neck flask equipped with a reflux condenser, a mechanical stirrer and a nitrogen supply. Then, the suspension was heated up to reflux and the reaction was allowed to proceed for 17 h under continuous nitrogen rinsing and stirring. Thereafter, the silica was washed three times with boiling methanol and boiling toluene each using a glass funnel of porosity 5 and dried in a vacuum chamber at 60 °C for 12 h.


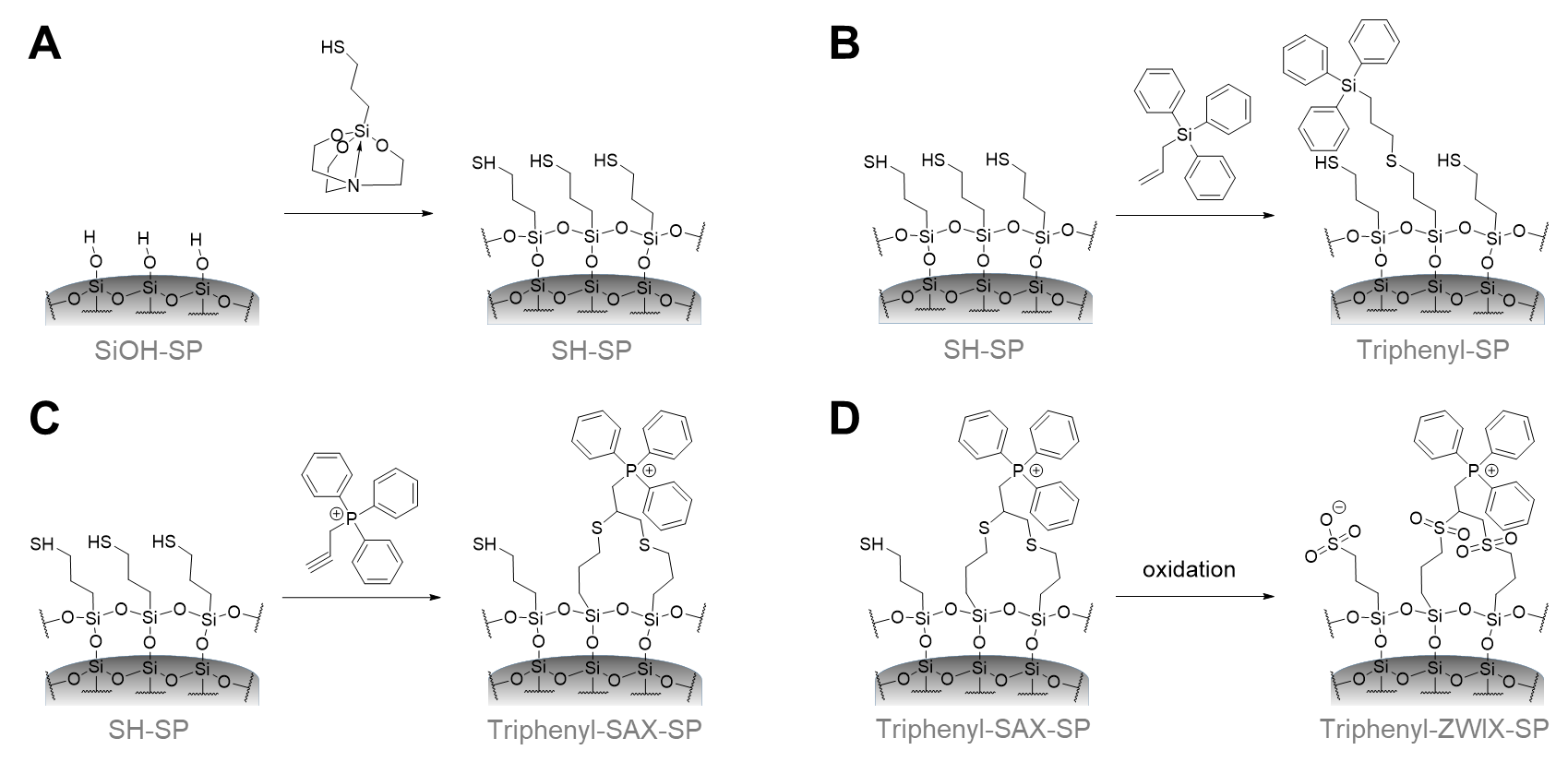


**Fig. S4.** Reaction schemes for the preparation of SH-SP (A), Triphenyl-SP (B), Triphenyl-SAX-SP (C) and Triphenyl-ZWIX-SP (D). Synthesis procedures can be found in the main document.

**Fig. S5.** Initial mechanism of silanol-catalyzed silica surface modification using (3-mercaptopropyl)silatrane. Due to the transannular donor-acceptor N→Si bond of the silatrane, the silatrane´s silicon atom is not prone to nucleophilic attacks, since it remains less polarized [1]. Nevertheless, the reactivity of the silatrane bumps up in acidic environments (such as silica surfaces) due to its oxygen atoms´ proton affinity (which is higher than for its nitrogen atom due to kinetic reasons [2]). By this means the formation of polarized hydrogen-bond complexes between the reactive acidic silanol groups on the silica surface and the Si-O bond of the silatrane becomes feasible making its linking to the surface in a concerted nucleophilic substitution reaction subsequently happen. During this reaction, the oxygen of the silatrane can also become protonated, making the silicon atom of the silatrane also susceptible to the subsequent nucleophilic attack of the silanol groups. However, both processes lead to the same outcome [3].


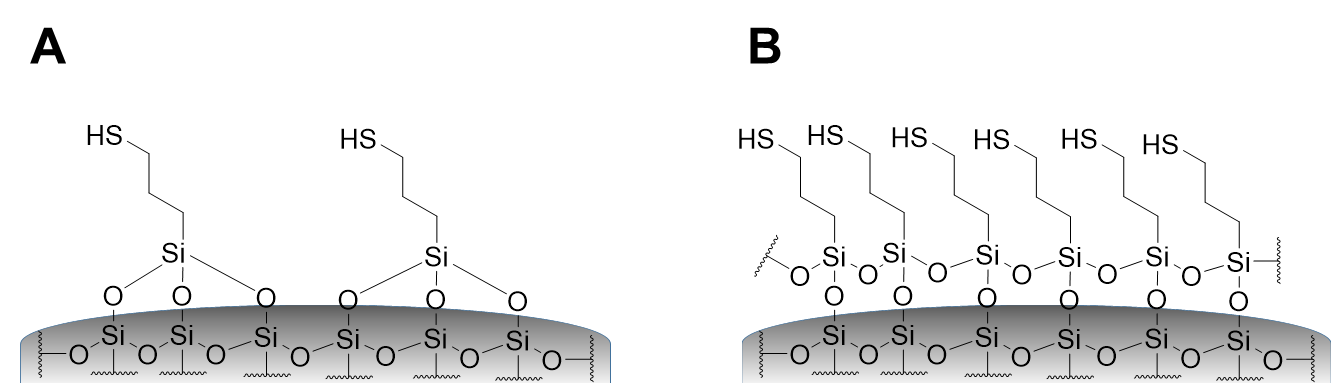


**Fig. S6.** Surface chemistries of silatrane-modified silica. There are two possibilities conceivable for the linkage of the silanization agents on the silica surface: brush-type trifunctional siloxane bonding (A) and polymeric siloxane bonding (B).


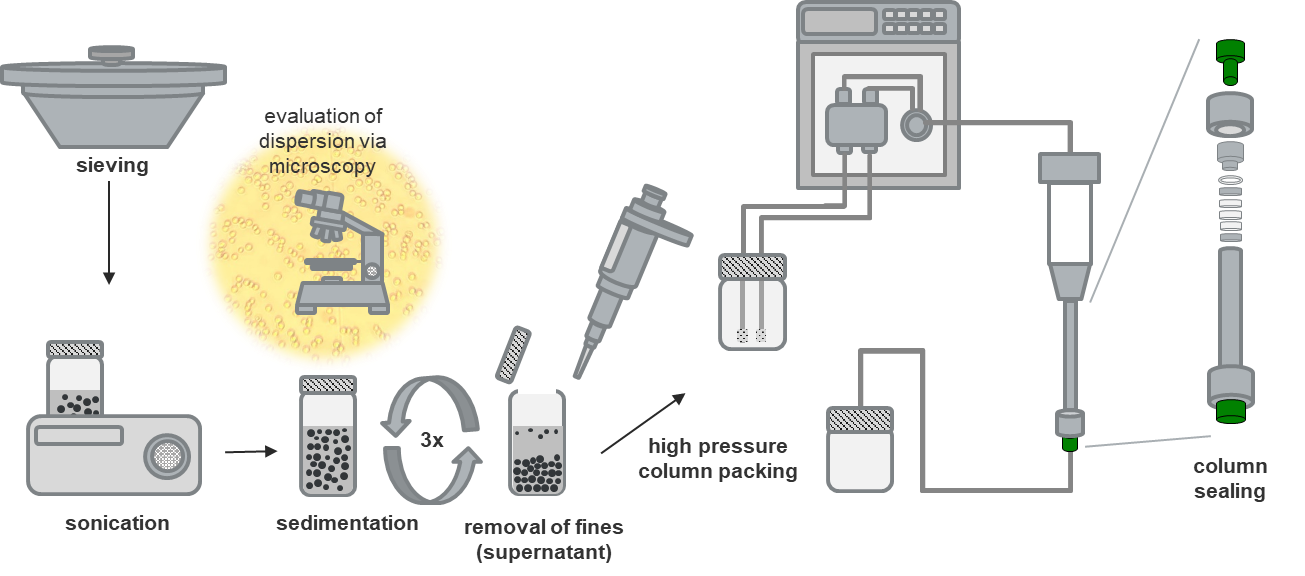


**Fig. S7.** General scheme of the in-house performed column packing procedure. The column packing procedure for analytical HPLC columns is a critical step in the preparation of analytical stationary phases. For the presented study, the prepared stationary phases were slurry packed into empty stainless-steel columns (50 mm x 3 mm) applying a pressure of 500 bar for 1 h. Beforehand, the slurry was prepared by suspending 250 mg silica in 5 mL 2-propanol. Methanol was used as pushing solvent. In the end, the column was rinsed for 24 h with methanol at a flow rate of 0.2 mL/min.

**PART III: Chromatographic tests and stationary phase classification**

**Table S1.** Chromatographic conditions of the Tanaka test for RP phases [4]. The hydrophobicity (test A) depends on the carbon load and surface area. It reflects the hydrophobic retention and surface area of the column. The methylene selectivity (test B) describes the ability of the stationary phase to discriminate analytes that differ in one methyl unit. The shape selectivity (test C) accounts for the capacity of a stationary phase to discriminate compounds of identical elemental composition but different three dimensional structure. Hydrogen bonding capacity (test D and E) depends on the amount of available silanol groups present on the phase and reflects the H-bonding capacity of a column. Ion exchange capacity at pH >7 (test F) reflects electrostatic attraction on the column, since at this pH most silanols possess a negative charge while benzylamine is positively charged. Ion exchange capacity at pH <3 (test G) reflects the number of acidic silanol groups on the column, since at this pH silanols are predominantly uncharged while benzylamine is protonated.

**
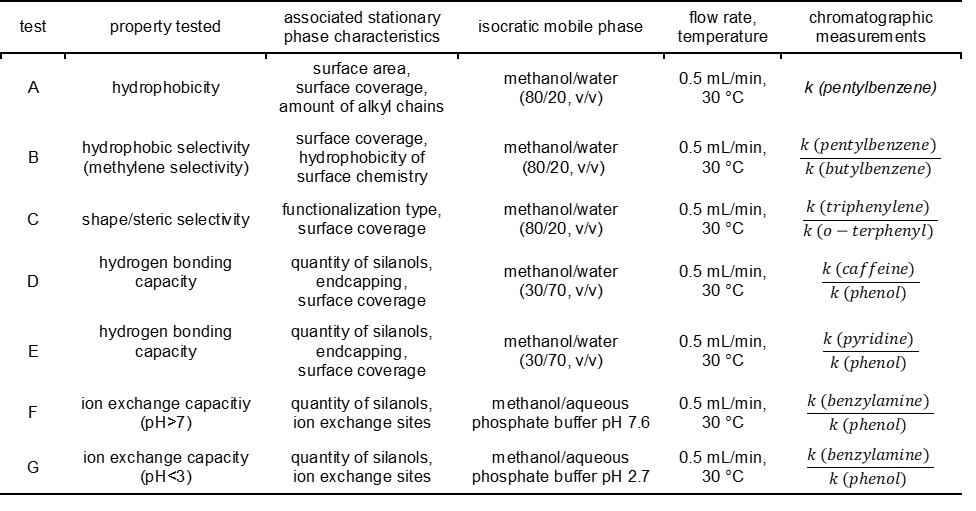
**


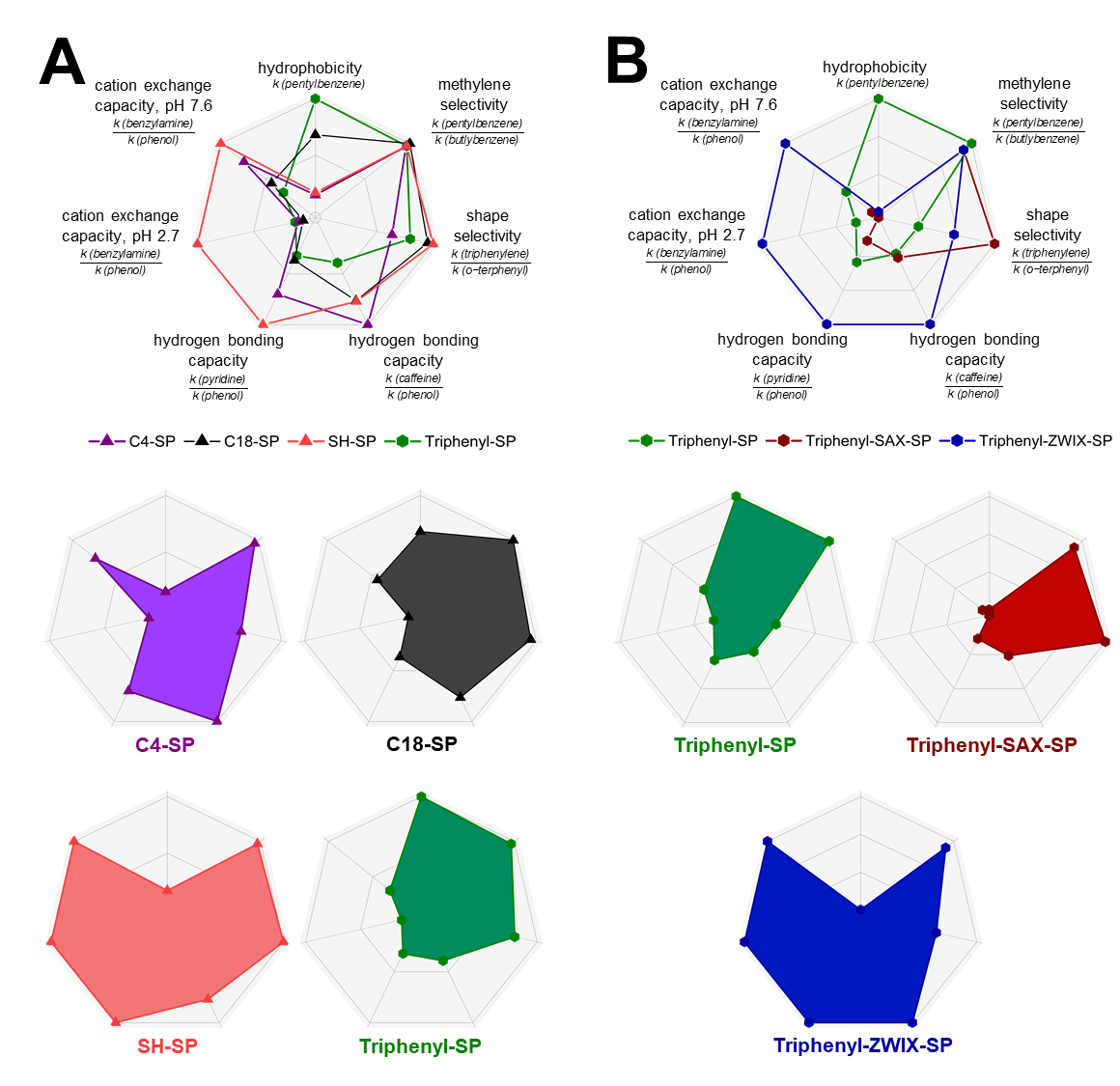


**Fig. S8.** Normalized radar plots obtained from Tanaka test. A: Comparison of the non-polar SPs (RP-type) C4-SP, C18-SP, SH-SP and Triphenyl-SP. B: Comparison of the three triphenyl-modified SPs Triphenyl-SP, Triphenyl-SAX-SP and Triphenyl-ZWIX-SP. In contrast to SH-, C4- and C18-SP, Triphenyl-SP exhibits less cation exchange properties at pH 7.6. This might indicate the efficient shielding of free silanol groups on the silica surface due to bulky phenyl groups diminishing the interaction between the silanols and the analytes.


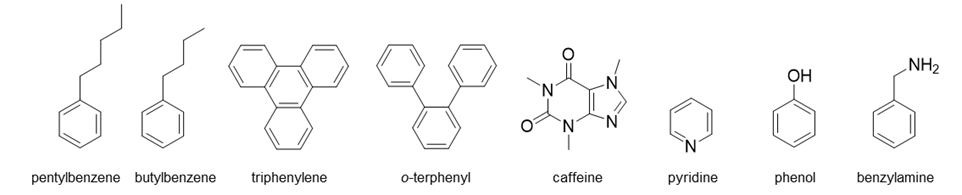


**Fig. S9.** Analytes applied in Tanaka test.

**Table S2.** Results of Tanka test.


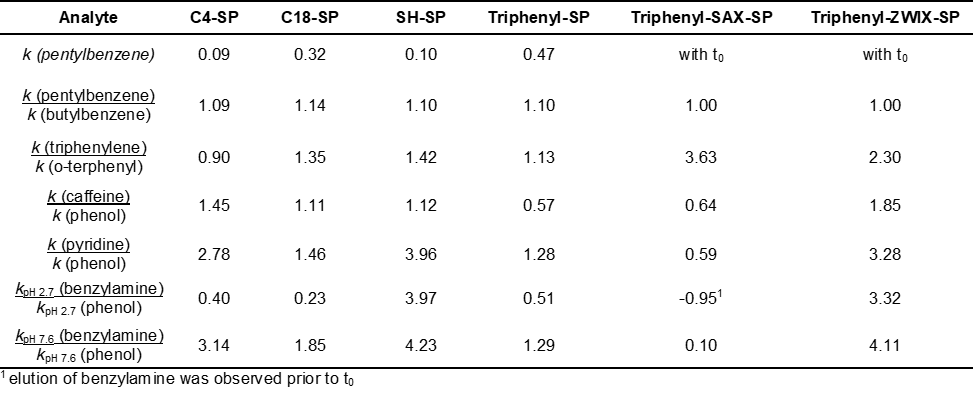


**Table S3.** Chromatographic data obtained from ion-exchange test. The separation of benzylamine, phenol and p-toluenesulfonic acid on Triphenyl-SP, Triphenyl-SAX-SP and Triphenyl-ZWIX-SP was investigated. Chromatographic conditions: Mobile phase consisted of MeOH/aqueous ammonium phosphate buffer (20 mM, adjusted to pH 3 or pH 7.5) (30/70, v/v), flow rate: 1 mL/min, temperature: 25 °C, injection volume: 5 µL.

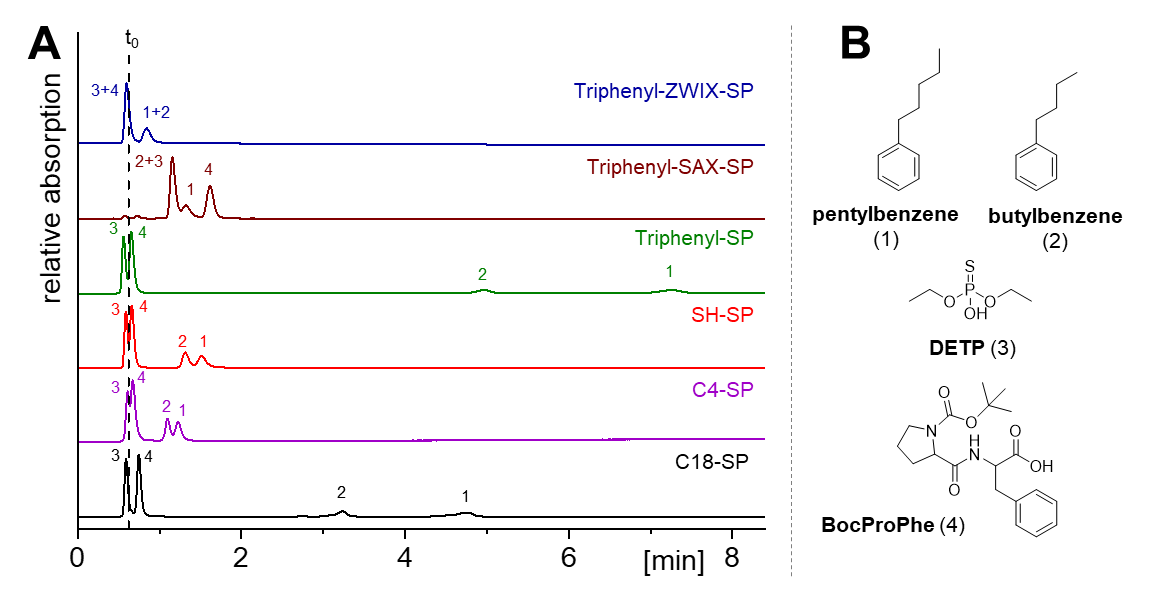


**Fig. S10.** Chromatograms obtained from RP test (A) and analytes applied (B). A mixture of ACN and water (40:60, v/v) containing 50 mM acetic acid was used as mobile phase. The pH of the mobile phase was adjusted to 6 utilizing ammonia. The linear flow velocity was set to 1.7 mm/s and detection was carried out at 220 nm. The injection volume was amounted to 2 µL and the temperature set to 25 °C. The analytes were all dissolved in the mobile phase reaching a concentration of 0.8 mg/mL. Uracil was used as void volume marker.


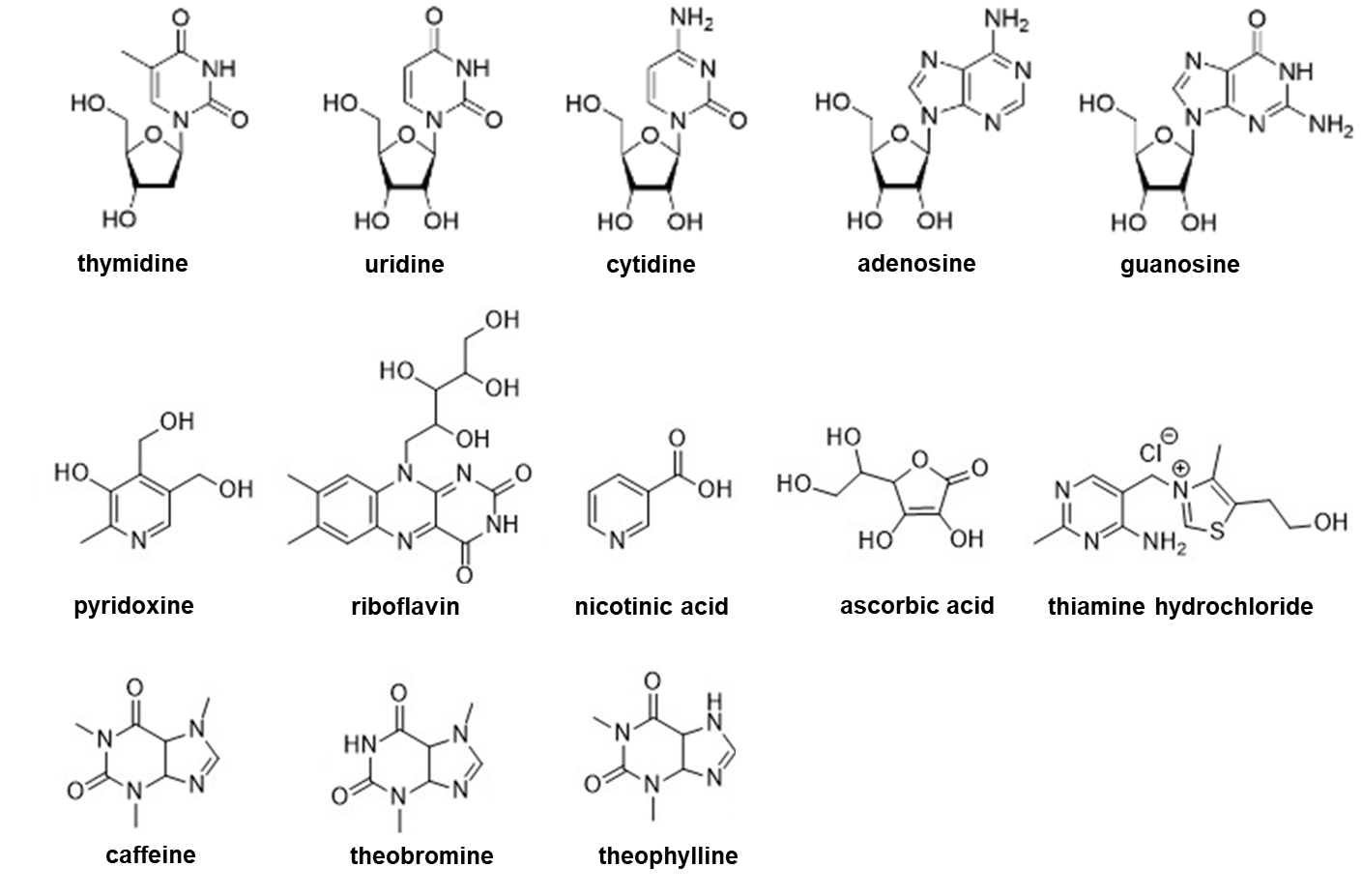


**Fig. S11.** Analytes applied for the HILIC tests. The chromatographic performance under HILIC conditions was investigated by the separation of xanthines (caffeine, theobromine, theophylline), nucleosides (adenosine, cytidine, guanosine, thymidine, uridine) and vitamins (nicotinic acid, pyridoxine, riboflavin, thiamine, ascorbic acid). The mobile phases consisted of ACN and water. The mixing ratio was 95:5 (v/v) for the xanthines and 90:10 (v/v) for the vitamins and nucleosides. Both mobile phases contained 5 mM ammonium acetate and showed an unadjusted apparent pH of 8 (measured in the hydro-organic mixture). The flow rate was calculated to the corresponding linear velocity of 1.7 mm/s. The void volume was determined by toluene.

**Table S4.** Retention factors obtained from RPLC and HILIC test. The retention factors listed for in-house prepared and commercial columns were used for PCA. Data of commercial columns were partially taken from [5] and [6].


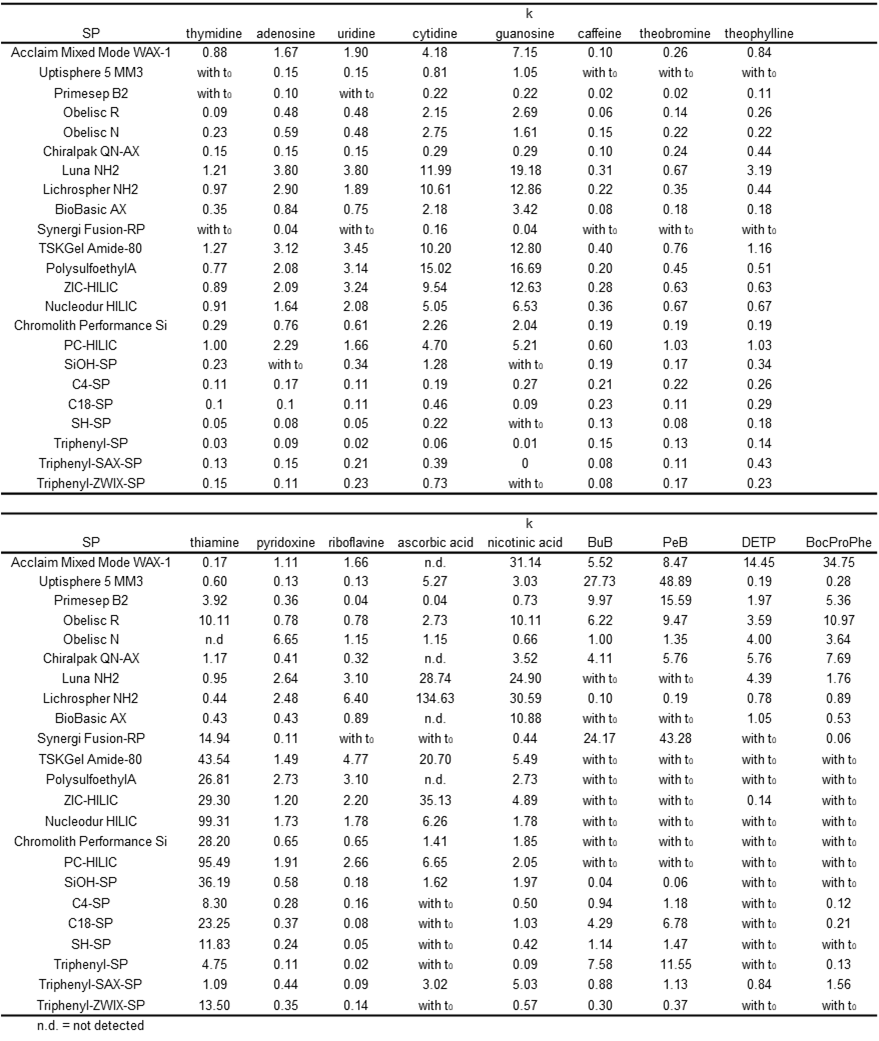


**Fig. S12.** Surface chemistries of commercial columns. In-house prepared columns C4-SP, C18-SP, SH-SP, Triphenyl-SP, Triphenyl-SAX-SP, Triphenyl-ZWIX-SP were compared to commercial columns based on the results from standard RP and HILIC tests via principal component analysis.


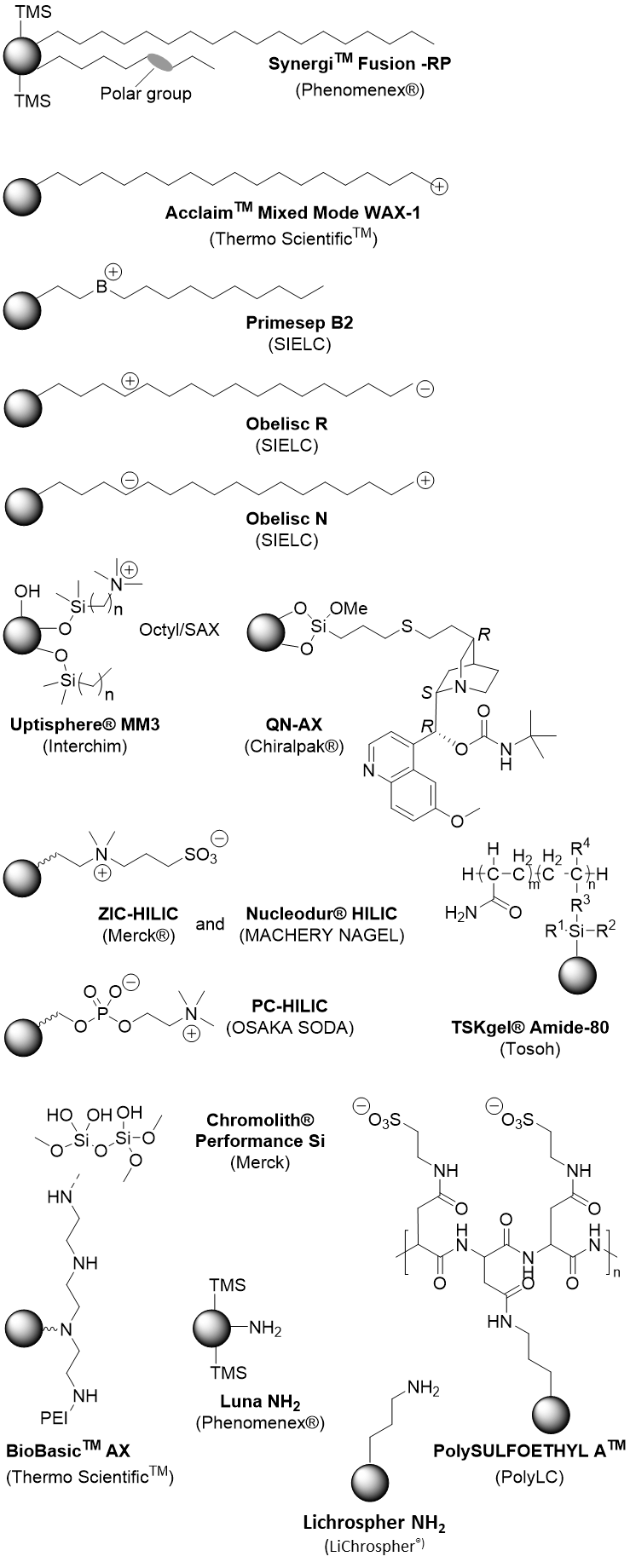

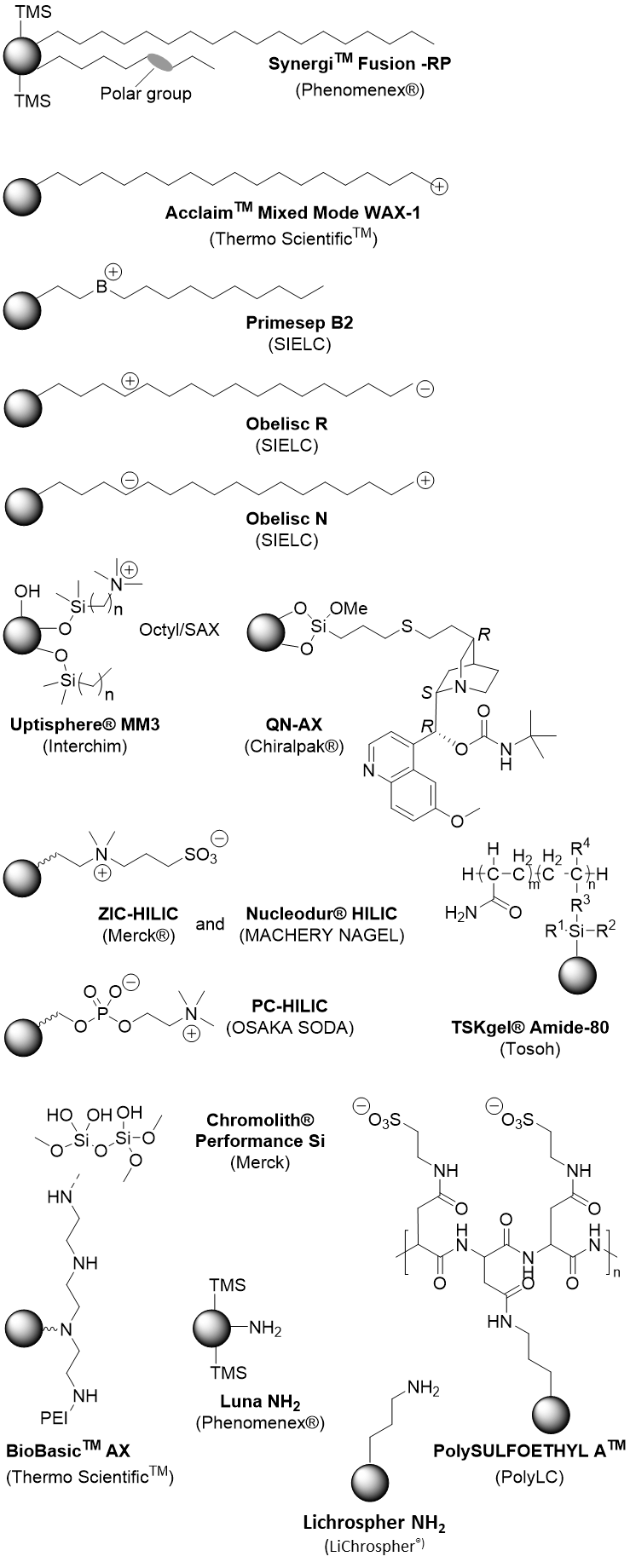

**Fig. S13.** Loadings scatter plot of principal component analysis: p1 vs. p2. p1 is the loading in the first component and p2 the loading in the second component. The loadings express the dominating correlation structure of the X matrix. Hence, p1 vs. p2 displays how the X-variables correlate to each other. The plot shows how the X-variables vary in relation to each other, which ones provide similar information, which ones are negatively correlated, or not related to each other, and which ones are not well explained by the model (p1 and p2 close to 0).


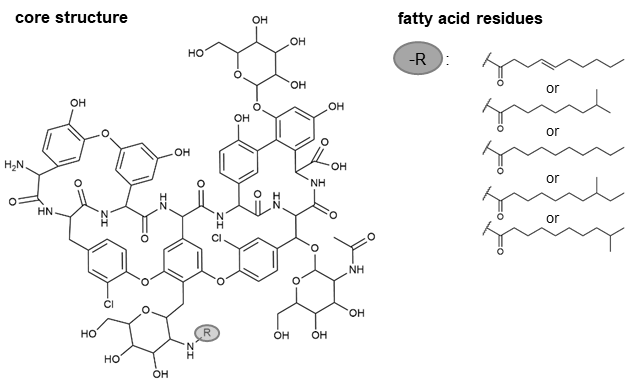


**Fig. S14.** Chemical structure of teicoplanin. Teicoplanin is a multicomponent antibiotic drug and consists of several glycopeptides varying in the attached fatty acid residue. [7]


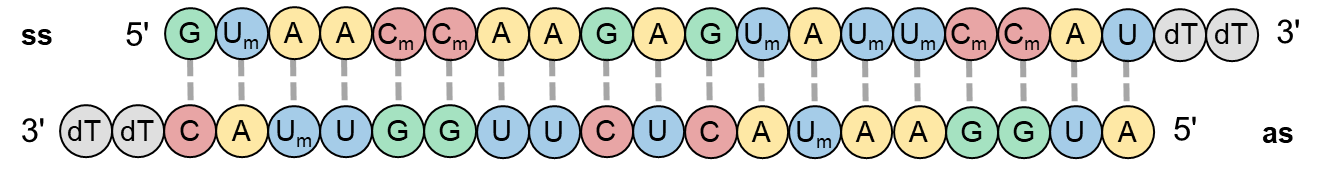


**Fig. S15.** Chemical structure of patisiran (ss: sense strand, as: antisense strand, dT: deoxythymidine, G: guanosine, C: cytidine, U: uridine, A, m: methylated). [8]


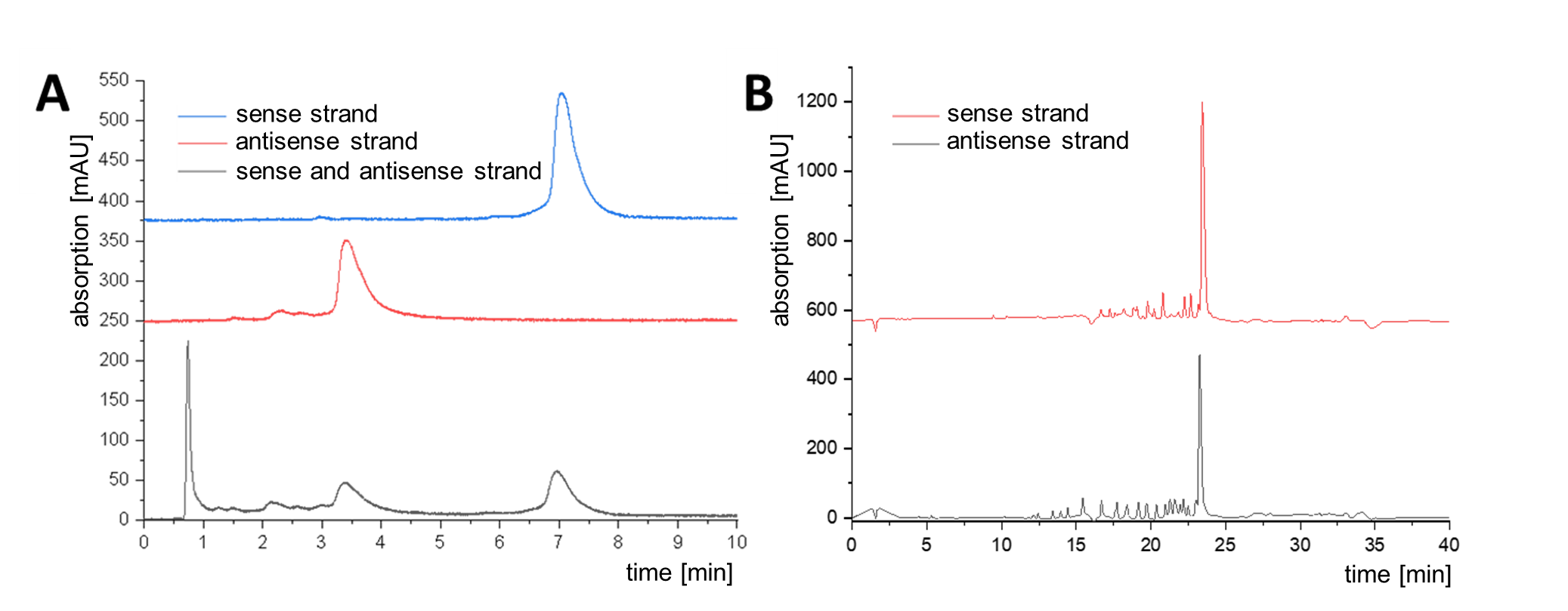


**Fig. S16.** Analysis of Patisiran (siRNA) strand and antisense strand. A: Analysis using in-house prepared Triphenyl-SP (50 x 3.0 mm, 3 µm, 300 Å). Chromatographic conditions: mobile phase A: aqueous 20 mM ammonium acetate, pH 6.8, mobile phase B: MeOH/water (9:1; v/v), containing 20 mM ammonium acetate, pH 6.8, gradient: 5% to 20% B in 10 min, temperature: 40 °C flow rate: 0.6 mL/min. B: Analysis using commercial BEH C18 column (50 x 2.1 mm, 1.7 µm, 130 Å,). Chromatographic conditions: mobile phase A: aqueous 100 mM tripropylammonium acetate, pH 7, mobile phase B: ACN/water (9:1; v/v), containing 100 mM tripropylammonium acetate , pH 7, gradient: 10 to 55% B in 32.5 min, temperature: 30 °C, flow rate: 0.3 mL/min.


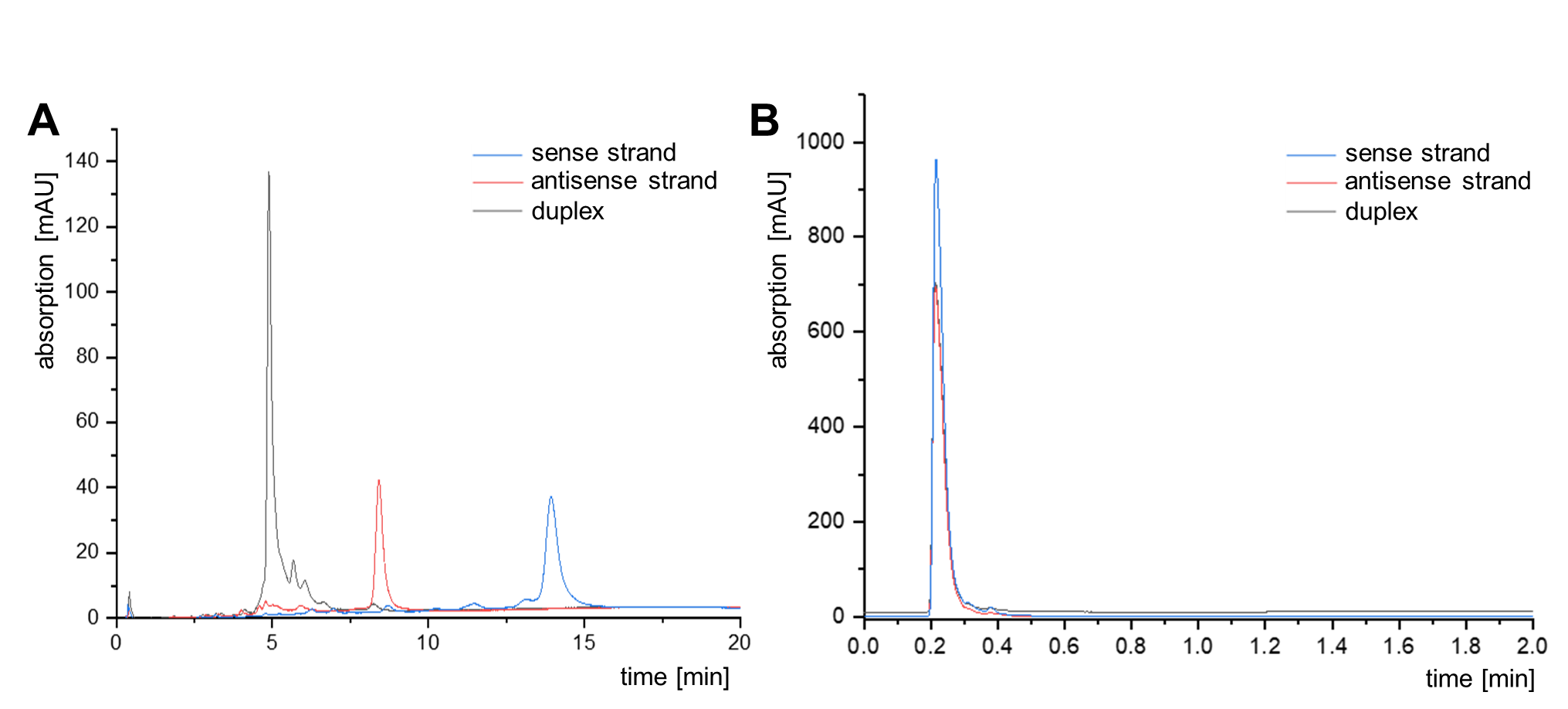


**Fig. S17.** Analysis of Patisiran (sense strand, antisense strand and duplex). A: Analysis using in-house prepared Triphenyl-SAX-SP (50 x 3.0 mm, 3 µm, 300 Å). Chromatographic conditions: mobile phase A: ACN/water (1:9; v/v), containing 20 mM phosphate buffer, pH 7, mobile phase B: ACN/water (1:9; v/v), containing 20 mM phosphate buffer and 1 M NaCl, pH 8; gradient: 0% to 50% B in 5 min; 50% to 100% B in 10 min, 100% B for 3 min, 0% B for 8 min, temperature: 50° C, flow rate: 1 mL/min. B: Analysis using in-house prepred Triphenyl-ZWIX-SP (50 x 3.0 mm, 3 µm, 300 Å). Chromatographic conditions: same as in A.

**PART IV: References**

[1] Puri JK, Singh R, Chahal VK. Silatranes: a review on their synthesis, structure, reactivity and applications, *Chem Soc Rev*. 2011; 40: 1791-1840.

[2] Sok S, Gordon MS. A dash of protons: a theoretical study on the hydrolysis mechanism of 1-substituted silatranes and their protonated analogs. *Comput Theor Chem*. 2012; 987: 2-15.

[3] Shlyakhtenko LS, Gall AA, Filonov A, Cerovac Z, Lushnikov A, Lyubchenko YL. Silatrane-based surface chemistry for immobilization of DNA, protein-DNA complexes and other biological materials. *Ultramicroscopy*. 2003; 97: 279-287.

[4] Kimata K, Iwaguchi K, Onishi S, Jinno K, Eksteen R, Hosoya K, Araki M, Tanaka N. Chromatographic characterization of silica C18 packing materials. Correlation between a preparation method and retention behavior of stationary phase. *J of Chrom Sci*. 1989; 27: 721-728.

[5] Wolter M, Geibel C, Olfert M, Su M, Bicker W, Laemmerhofer M. Development and chromatographic exploration of stable-bonded cross-linked amino silica against classical amino phases. *J Sep Sci*. 2022; 1-15.

[6] Zimmermann A, Horak J, Sanchez-Munoz OL, Laemmerhofer M. Surface charge fine tuning of reversed-phase/weak anion-exchange type mixed-mode stationary phases for milder elution conditions. *J Chromatogr A*. 2015; 1409: 189-200.

[7] Geibel C, Olfert M, Knappe C, Serafimov K, Laemmerhofer M. Banched medium-chain fatty acid profiling and enantiomer separation of anteiso-forms of teicoplanin fatty acyl side chain RS3 using UHPLC-MS/MS with polysaccharide columns. *J Pharm Biomed Anal*. 2023; 115162.

[8] Li F, Chen S, Studzinska S, Laemmerhofer M. Polybutylene terephtalate-based stationary phase for ion-pair-free reversed-phase liquid chromatography of small interfering RNA. Part 2: Use for selective comprehensive two-dimensional liquid chromatography. *J Chromatogr A*. 2023; 464069.
